# A Novel approach to ship valuation prediction: An application to the supramax and ultramax secondhand markets

**DOI:** 10.1371/journal.pone.0319073

**Published:** 2025-05-14

**Authors:** Elif Tuçe Bal, Ercan Akan, Huseyin Gencer

**Affiliations:** 1 Department of Maritime Business Administration, Iskenderun Technical University, Hatay, Turkey; 2 Department of Maritime Transportation Management Engineering, Iskenderun Technical University, Hatay, Turkey; 3 Department of Maritime Business Management, Piri Reis University, İstanbul, Turkey; University of Madeira / NOVA Lincs, PORTUGAL

## Abstract

Accurate ship valuations are very important in ship sales and purchase (S&P) transactions and for marine insurance purposes. It is equally important to select an appropriate valuation methodology. Today, one of the methods is Machine Learning (ML) algorithms stand out in generating better results than traditional methods. The aim of this study is to propose a highly accurate ship valuation model for the supramax/ultramax segment to interested parties using ML methods, with models established on the basis of linear regression. For this purpose, a four-stage path was followed. (i) The first data set, in which the significance of independent variables for supramax/ultramax ships was tested and linear regression models were created with statistically significant variables, covers the period from August 2005 to December 2022. At this stage, a model was first created that takes into account the values of the Baltic Exchange indices in the month of sale as an independent variable, and then another model that takes into account the values in the month of sale and the values in the months before the month of sale as independent variables. (ii) For the two linear regression models created; Price predictions were made with Linear Regression, Decision Tree, Random Forest and XGBoost ML algorithms. (iii) In the next stage; the two models created were simplified to include independent variables that can be easily obtained from the market in the model; and the obtained simplified models were re-predicted with ML algorithms. The fact that the first model and the simplified model are close in terms of prediction performance shows that the simplified model can be used in prediction. (iv) In order to show that the simplified model can produce reliable results when the data set is expanded, 2023 data was added and price predictions were made again with ML algorithms. As a result, the simplified model’s predicting performance was further improved with the addition of new data; the model established with the Baltic Exchange indices in the months before the sales month provided a significant superiority over the other model. XGBoost stood out as the best method according to performance criteria.

## 1. Introduction

The advantage of transporting large amounts of units at once and the economy of scale gained thanks to this advantage make maritime transportation the most preferred mode of transportation in international transportation. Ships with high investment amounts constitute the most important unit of maritime transportation, where 12,027 million tons of cargo were transported in 2022 [1]. As of January 1, 2023, there are 56,591 ships of 1,000 gross tonnage (GT) and above in the world, and the commercial value of these ships reaches 1.26 trillion dollars [1]. The total commercial value of dry bulk carriers is 298 billion dollars as of January 1, 2022 [2]. Over the years, most of the ships in the fleet either find a place for themselves in the secondhand market, are subject to banks due to loans, are included in the accounts of insurance companies to guarantee themselves, or are included in all levels of a company because they are operated for a purely commercial purpose. During these processes, the ship comes into existence not only with its physical existence but also with the commercial value of its physical existence, and the commercial value of the ship becomes even more important at this point.

One of the most important reasons for changes in ship owners’ overall cash flows is the changes in the value of the asset they own, namely the ship [3]. Changes in shipping markets, where short-term cycles dominate, are also reflected in ship values. For example; a new VLCC, which was cheaper than $40 million in 1986, could be sold for $90 million just 4 years later. In an environment where ship prices change so rapidly, relevant parties need to check the real values of the asset in question [4]. Shipowners benefit from such high price changes by buying and selling ships. This asset game has a greater impact on the balance sheet than the operation of the ship. One of the points to be noted here is that the ship, as an asset, is in competition with other assets. Since ships are in competition with other assets, businesses make investment decisions by comparing the return of the ship with the return of other assets [3]. Accurate estimation of ship values plays a key role in making decisions about keeping, selling, or buying a new ship. Ship brokers are at the center of ship valuation. While the general practice is to determine ship values through comparison based on recent sales; In the absence of recent sales, ship values reflect the prejudices of ship brokers. In order to overcome broker biases, it is preferred to obtain prices for a ship from several different brokers and use the average of these prices as the ship value [4].

According to Stopford [4], ship valuation has five common areas of use: (i) determining the market value of the ship purchased or offered for a loan; (ii) determining the current value of the ship in order to determine whether it meets the loan agreement in case the ship is held against collateral value due to loan terms and to protect financial guarantees [5]; (iii) determining the market value of the fleet of a company offering a public offering or issuing bonds; (iv) a company publishing its accounts determines the value of its fleet; (v) When there are not enough ships sold in the secondhand market, the secondhand ship investor determines the market value in order to check the price [4]. In addition, shipowners for accounting, planning, and control; while potential ship buyers need valuation in their investment decisions and sellers when selling the ship [6]; Furthermore, valuation is also very important for brokers, financial resource providers, insurers, and investors. Accurate ship valuation; while enabling more consistent decision-making for all parties in terms of lending capacity, allocation of portfolios, stock pricing, and risk management [7]; also in accurately determining the insurance value of the ship [8]; It enables managers to make reliable planning and forecasting in implementing capital-intensive ship investments, designing alternative financial resources, and controlling risks [9].

Ships are crucial capital assets for ship owners, and their valuation is crucial for determining their business’s capital structure. They use the majority of the capital and are subject to annual revaluation, which is essential for credit agreements. International Valuation Standards Council [10] provides three basic valuation approaches: market, income, and cost. The market approach calculates the value of an asset by comparing it with similar assets, while the income approach converts future cash flows, income, or cost savings into present value. The cost approach calculates the current replacement cost or reproduction cost of the asset and deducts physical deterioration and wear and tear. The market approach is the most preferred in the shipping industry, with three steps: determining factors for comparison, determining a sufficient number of reference ships, and determining the ship’s value. The income approach considers the present value of future cash flows, using the weighted average cost of capital (WACC) as a discount. The cost approach is used for uniquely functional or special ships, adjusting the replacement cost by considering physical wear and functional obsolescence [11,12]. This study presents a high accuracy model to obtain the best results in forecasting using the market approach.

Ships that are the subject of studies in the literature; It is involved in studies either with different ship types, either according to a specific ship type (tanker, dry bulk, container, etc.), or according to a specific segment of a specific ship type (e.g., capesize ships under dry bulk carrier). Studies reveal that there are differences in the volatility of freight rates [13], the variability of secondhand prices [14,15], and the increase or decrease of risks [16] between the same type of ships in different segments. Furthermore, studies categorizing results based on ship types and sizes demonstrate that findings vary according to these distinctions, highlighting the necessity of such categorization [8,17–20]. There are studies in the literature that take into account a certain ship type and size, and these studies are becoming more common [7,11,21–29]. Studies aimed at determining the price of ships or the factors affecting the price of ships, where the size of a particular ship type is taken into account and individual ship sales are the dependent variable; it has concentrated especially on dry bulk carriers and handysize size with the idea that the sample could be larger [7,23,25,28,30]. There are also studies considering Capesize and Panamax sizes in the dry bulk carriers field, and chemical tankers and VLCCs in the tanker market [11,24,26,27,29].

ML, which encompasses statistics, artificial intelligence, and computer science, is also known as predictive analytics or statistical learning. Today, ML methods are widely used in many fields and have a high impact on the way data-driven research is conducted [31]. ML methods stand out with their ability to process big data quickly, produce better solutions to complex problems than traditional approaches, and obtain insight about big data [32]. ML methods, being among the modern real estate valuation methods, have started to be employed in the valuation of ships, which are real estate. In studies conducted by Lim et al. [26], Adland et al. [7] and Kim et al. [29], where method comparisons were made, it was seen that the prediction performances of ML methods surpassed traditional regression methods.

According to the 2020 data of the United States Department of Agriculture [33], there are 2085 and 1039 ships belonging to the Supramax and Ultramax classes, respectively, and these two classes constitute 25% of the total dry bulk carriers. When examined in terms of capacities, the capacity for Supramax ships is 115.8 m.dwt; while for Ultramax ships it is 65 m.dwt. The total capacity of the two classes constitutes approximately 20% of all dry bulk carriers. Developments in shipbuilding technologies that allow the construction of more efficient and larger ships contribute to the increase in the carrying capacity of the ultramax ship fleet in particular [34]. According to AXSData [35]; While the utilization value per commodity of Ultramax vessels in 2023 is 444.5m MT, that of Supramax vessels is 630.5m MT. Compared to the previous year, there was a 14.2% increase in Ultramax vessels and a 7.6% increase in Supramax vessels. When the sales under dry bulk carriers reported on the website of CW Kellock and Co ship brokers since 2008 are examined; In 2023, from Supramax/Ultramax ships reflecting the range of 50,000–68,000 DWT according to Baltic Exchange [36]; It is seen that 207 units were sold and since 2017, Supramax/Ultramax ship sales constitute 30% of total dry bulk carrier sales [37]. Therefore, these values also show how large the transaction volume of Supramax/Ultramax ships is. The variability of the results according to the segments under a ship type is due to the fact that the studies in the literature increasingly focus on samples based on a segment. The idea that studies in the DWT range under certain ship types, especially in certain segments, will increase the consistency and accuracy of ship valuation has led to the idea of applying the study on a ship type segment. There are no studies on Supramax/Ultramax ships in the literature. Considering the sales volume, it is thought that a practical valuation model for Supramax/Ultramax ships will be useful for ship buyers and sellers, financing providers, insurers, and managers. With this motivation, the study was conducted on the valuation of Supramax/Ultramax ships, which is a dry bulk carriers segment.

Under this motivation; *The aim of the study is to propose a highly accurate ship valuation model for the supramax/ultramax segment, which will be available to interested parties in the market, using machine learning methods, based on linear regression models created with variables determined by testing their significance on the basis of linear regression.* Enables objective evaluations rather than subjective evaluations; It can be easily used by brokers, shipowners, financing providers, insurers, and all units that need ship valuation; A ship valuation model with high accuracy will benefit the relevant parties. In addition, it is thought that an easily applicable model will contribute to the relevant parties in terms of price control in case of receiving ship valuation services from third parties.

The study provides a high-accuracy model for the valuation of supamax/ultramax vessels using data that can be easily extracted from broker reports and ML methods that practitioners can easily implement in programs such as Python and R; and it also contributes theoretically by testing the statistical significance of P&I and flag variables, which have not been included in previous studies, on supramax/ultramax vessel prices. In the study, a comparison was made between the model that includes the values of Baltic Exchange indices in the month of sale and the model that includes the values of Baltic Exchange indices in the months before the month of sale. As a result of the comparison, it was revealed that the model that includes the values of Baltic Exchange indices in the months before the month of sale produced much better results. Using the Baltic Exchange indices in the months before the month of sale instead of the Baltic Exchange indices in the month of sale allows practitioners to make ship price predictions several months in advance.

The rest of the study is designed as follows; while there is a literature review on general variables and models for ship valuation in the second chapter; description of the methods to be used in the study and statistical information about the data sets in the third chapter; the fourth chapter includes the results of method applications, results, and discussion with the studies in the related literature in the fifth chapter, and with conclusion, limitations and future research by the final chapter.

## 2. Literature review

In this section, first of all, studies on ship valuation are discussed from a general perspective. The variables and models used in ship valuation are examined in detail under subheadings.

The first of the models created for ship prices in the literature is the average price of a 70,000 DWT dry bulk ship; It was made by Charemza et al. [38], who linked it to the index of time charter rates, the world heavy industry production index, and a dummy variable expressing the effect of the energy crisis. The study also includes equations for the average prices of OBOs. Beenstock [39], who put forward another equation for ship prices, argued that ships should be considered as capital assets and explained with capital theory while taking into account the theoretical foundations of ship prices. Capital theory examines models of economic transformation, illustrating the relationship between present economic choices and future output levels, while demonstrating the interconnections among various components of economic theory, such as production, demand, and distribution, within a dynamic framework. In capital theory, comprehending the temporal growth of capital is essential. Investors acquire assets with the intention of achieving capital gains by selling them for a price beyond their original acquisition cost [40,41]. In this respect, according to capital theory, the future value of ship prices reflects the demand for ships. The researcher calculated the expected return of the ship by taking into account net income and capital gain, stating that ships, which are capital assets according to ship owners, are in competition with other capital assets. The method used by the researcher is similar to the income method among valuation methods. Beenstock [39] conducted this study under the acceptance of the Rational Expectations Hypothesis. Beenstock et al. [42–44] applied the theoretical study by Beenstock [39] to both the dry bulk market and the tanker market. They also implemented the theory by integrating the dry bulk market and the tanker market. In the studies, under the assumption that investors have rational expectations; A model has been created in which freight rates, laid up, new and secondhand ship prices and fleet size are determined together and dynamically. The acceptance of the Rational Expectations Hypothesis in the studies conducted by Beenstock [39], Beenstock et al. [42,43] points to the efficiency of the market and raises the question of whether markets are efficient. While the efficiency of the market is related to the ship price containing all available information [45]; The inefficiency of the market creates arbitrage opportunities such as purchasing ships at lower prices or selling them at higher prices than their fundamental value, which is calculated by reducing the value of the possible future income of ships to their current value [46]. In the literature, the efficient market hypothesis has been discussed by many researchers in order to measure whether the market is efficient or not [45–50]. Hale et al. [45], who answered the question of market efficiency based on the assumption in Beenstock’s [39] study, suggested that the efficient market hypothesis may not be valid for dry bulk carriers. Glen [47], who developed the work of Hale et al. [45] with a more modern method, stated that if the common trends are of stochastic origin, cointegration will not express the inefficiency of the market; Considering this situation, he argued that long-term market efficiency is valid. Kavussanos et al. [46], who tested the efficient market hypothesis based on newbuilding and secondhand ship prices in the dry bulk market, rejected the efficient market hypothesis for both. With the results obtained, Adland et al. [48] demonstrated the validity of the efficient market hypothesis for bulk carriers in the secondhand markets. The study conducted by Engelen et al. [50] also reveals that efficiency in dry bulk shipping is maintained in the short term.

In addition to studies measuring the efficiency of the market, there are also studies in which the variables affecting secondhand ship prices are determined by time series [17,19–21]. In the time series approach where the variability of secondhand ship prices is the subject, Alizadeh et al. [18] and Syriopoulos et al. [51] examined the relationship between transaction volume and the fluctuation of secondhand ship prices. Adland [22]; Taking the rise in the freight market between 2003 and 2005 at the center of the research; examined the relationship between the secondhand market, freight market and newbuilding market and revealed the existence of a close relationship between the secondhand market, freight and newbuilding markets. Merikas et al. [52] used the secondhand ship price/newbuilding price ratio in investment decision modeling. In another study including secondhand ship prices, Lun et al. [53]; examined the relationship between newbuilding prices, secondhand ship prices, demolition prices, fleet size, freight rates and maritime trade. In the study conducted by Adland et al. [54], the secondhand market is included together with the four main maritime markets (newbuilding market, secondhand market, demolition market and freight market); A single-equation mathematical relationship describing simultaneous equilibrium prices has been established.

With Adland et al. [23], studies in which individual ship sales are the dependent variable began to appear in the literature. Adland et al. [23], Köhn [24], Kalouptsidi [30], Albertijn et al. [11], Adland et al. [25], Merika et al. [55], Lim et al. [26], Adland et al. [27], Junior et al. [56], Adland et al. [7], Peng et al. [28], Kim et al. [29] followed.

### 2.1. The variables utilized in ship valuation studies

According to the equation established by Beenstock [39], ship prices are positively related to world maritime trade, scrap value, fuel prices and world wealth; It is negatively related to the return on the competing asset and the laid-up cost of the ship. While Tsolakis et al. [17] and Haralambides et al. [19] determine secondhand ship prices, they use newbuilding rates, time charter rates and order size (orderbook/fleet) as market indicators; and 3-month LIBOR value as capital cost and they used a dummy variable to avoid problems with outliers. The researchers’ expectation is that secondhand ship prices are positively related to freight rates and newbuilding rates, while they are negatively related to LIBOR. According to researchers, order size can affect secondhand ship prices positively or negatively. Wright [21] divides the factors affecting the secondhand ship price into market fundamentals, which indicate supply and demand relationships, and market sentiment, which indicates short-term speculations; It has been concluded that both market fundamentals and market sentiment are effective on secondhand prices in the long term, while market fundamentals are effective in the short term. Thalassinos et al. [20], who built a valuation model for secondhand ship prices using time series, added the margin variable to the LIBOR variable, unlike the studies in the literature. According to researchers, the possible impact of LIBOR and margin variables on secondhand prices is negative. Another point that distinguishes the research from other studies is that they add operating profit as a variable instead of time charter rates as a market indicator. The variables included in the study are; newbuilding prices, industrial production, order/fleet ratio, and scrap value. While possible expectations are positive for operating profit, newbuilding prices, and industrial production; It can be positive or negative for order/fleet ratio and scrap values.

Adland et al. [23], who used independent ship sales as dependent variables for the first time in their analysis of Handysize ships, added DWT, age, and freight market variables to the model as independent variables. Researchers have stated that these three variables alone may not explain the price, and ship-specific factors such as fuel consumption, shipbuilding country, and machinery manufacturer can be added to the model. Köhn [24], who researched chemical tanker sales; In addition to DWT, age, daily earnings, newbuilding price, builder country, the classification society, vessel speed, and ice class variables; specific to chemical tanker; He added hull type, coating, number of tanks, IMO type, cargo diversity (the interaction term between the number of cargo tanks and number of different available coatings), and pump diversity (the interaction term between the number of pumps and pump capacity) variables. Pruyn et al. [57], with the perspective developed by examining the 20 years between 1991 and 2011; In models developed for price, a minimum number of independent variables should be taken into account, such as newbuilding price level, order size, profit (or earnings and fuel/fuel consumption costs), age and DWT; and stated that variables such as speed and horsepower (HP) can be added to these independent variables. Kalouptsidi [30], whose main aim is to establish and predict a dynamic model for entry and exit into the maritime market; used secondhand sales as the value function in the model. The independent variables in the value function include the age of the ship, the age distribution of the fleet, the time required to produce and deliver the ship, and the total demand for transportation services. Mietzner [6], who conducted a price determination study for container ships based on the model he created; He added depreciation, the fact that the sales to be used in the comparison were made close to the date of valuation, TEU capacity, the number of reefer plugs for the refrigerated container, and the presence of a crane on the ship as variables to the model. Albertijn et al. [11], who built a valuation model on Capesize ships, used the Baltic Capesize Index as an independent variable, which is the first study in which the index was used as an independent variable in ship valuation. In the study alongside BCI; age, DWT, dummy variable to express closeness of sale date; and the interaction term, which includes age together with the dummy variable expressing the age-price relationship, was used. The study by Adland et al. [25], whose main basis is on the effect of energy efficiency of ships on ship prices, was conducted on handysize ships. In the study, researchers used the ship’s fuel consumption and the fuel efficiency index as independent variables expressing energy efficiency. The study also included variables thought to affect the price of the ship: One-year time charter rate, DWT, capacity of cranes in tons, speed, builder country, buyer country, and a variable expressing how many times the ship was sold before the last sale. Another study in which the fuel efficiency index is the independent variable was conducted by Ross [58] on Container, Dry Bulk, Tanker, RoRo – RoPax - Ferry, Gas Carrier ships. In the study, the effect of the fuel efficiency index on price variability was examined by using the market change compared to the previous year as the dependent variable. In the study conducted by Merika et al. [55], where the logarithm of individual ship sales is the dependent variable; Variables affecting ship price heterogeneity were investigated using age, 3-month LIBOR, annual time charter rates, ship size, newbuilding prices, scrap values, dummy variable reflecting technology, and the independent variable of fleet size as a supply-side factor. As a result of the research, it was revealed that age, 3-month LIBOR and annual charter rates were effective in ship price heterogeneity. In the study conducted by Lim et al. [26] on panamax ships, although freight, newbuilding price, order, scrap value, age and DWT were determined as independent variables by literature review; After the correlation and stepwise regression, freight, age and DWT were selected as independent variables. The study by Junior et al. [56], which basically measured the price impact of the countries where ships were built; It has been applied on tankers, bulk carriers, and container ships. In addition to dummy variables of shipbuilding countries, DWT, age, newbuilding prices, LIBOR and earnings indexes were used in the study. In the study where a separate index was used for each ship type, the Baltic Dry Index was used for dry bulk ships. The study conducted by Adland et al. [7] to investigate the effect of the ML technique XGBoost on secondhand ship valuation was based on handysize ships. As independent variables in the research, DWT, age, speed, fuel consumption, main engine horsepower, rpm, cubic utilization, total crane capacity, fuel efficiency index, main engine manufacturer, builder country, fuel type, number of hatches and holds, one-year time charter rate, order/fleet rate, and LIBOR were used. In the study where variable selection was made with the Boruta algorithm, the first five variables in order of importance were determined as age, one-year time charter rate, fuel efficiency index, order/fleet ratio, and DWT. In the study on handysize ships by Peng et al. [28], where the main research subject is the effect of investor domicile on secondhand ship prices; One-year time charter rate, age, DWT, design speed, main engine fuel consumption, horsepower, builder country were used as independent variables. In the study, the effect of investor domicile is considered as both buyer-side and seller-side, as well as the matching of buyer and seller countries. In the study conducted by Kim et al. [29] on very large crude carrier ships, VLCC freight, vlcc supply, VLCC scrap price, VLCC newbuilding price, VLCC order/supply rate and LIBOR were used as independent variables. In the study conducted by Nam et al. [8], whose main subject is to contribute to the newbuilding industry with managerial implications and to determine the factors affecting the prices of secondhand ships; Bulk dry carriers, tanker and container ships were studied and age, size, freight, whether the ship was sold under a time charter agreement, builder country, and type of main engine were taken into account as independent variables. Size, freight, and age were used as control variables in the study.

### 2.2. The models utilized in ship valuation studies

In studies on ship valuation in samples of individual ship sales, methods can be grouped under two main headings: those used to determine the effects of independent variables or to estimate ship values. Multivariate Density Estimation (MDE) [23], Generalized Additive Model (GAM) [24,27], Hedonic price regression [25], Nonparametric Regression [28,55] and Quantile Regression [55], Linear Regression [8,28,56,58] are among the models used to investigate the effects of independent variables on ship values.

In the studies, GAM [11] and Linear Regression [11,29] were used to determine the effects of independent variables on the dependent variable, as well as to make predictions. Apart from these models, Stepwise Regression [26], Artificial Neural Networks [26,29], LASSO regression [7], General Linear Model (GLM) [7], XGBoost [7], Random Forest Algorithm [29] are also among the models used in estimation.

Lim et al. [26], who compared Stepwise Regression and Artificial Neural Networks in their study, revealed that Artificial Neural Networks make better estimations than Stepwise Regression. In the study conducted by Adland et al. [7] comparing LASSO Regression, GLM, GAM, and XGBoost, the best estimating model was XGBoost, followed by GAM, GLM, and LASSO Regression. In the study of Kim et al. [29], who made estimations with Linear Regression, Random Forest, and Neural Network Regression, the best model was Neural Network Regression, followed by Random Forest and Linear Regression. In the study including sub-models for Random Forest and Neural Network Regression models; Although the best model according to RMSE values was established with the sub-model of Neural Network Regression, close results are obtained in other sub-models of Neural Network Regression and Random Forest. In the studies conducted by Lim et al. [26] and Kim et al. [29], it was also intended to make predictions in this study with Neural Network Regression, which achieved the best results, but it was not added to the compared models because the results obtained according to the choices were very variable; Neural Network Regression was weaker than the preferred methods such as XGBoost and Random Forest, and it was thought that it would not be practical in practice in this study, which was especially aimed at practitioners. Linear Regression was added to clearly demonstrate the model improvement in the study, and it was followed by Decision Tree, which forms the basis of XGBoost and Random Forest. Unlike Linear Regression, XGBoost and Random Forest can reveal non-linear relationships between dependent and independent variables, and this is effective in producing better results than linear regression. While XGboost combines weak decision trees and achieves higher accuracy compared to the decision tree model; Random Forest reduces the risk of over-fitting by combining decision trees. Based on these reasons; XGBoost and Random Forest, which have achieved good results in many studies in the literature, have been added to the compared models with the idea that the best estimation can be obtained with these methods. Apart from the known methods, there are also methods designed by researchers [6,59].

Although methods such as XGBoost and Random Forest are included in the ship valuation literature, this article is the first time that they are used for Supramax/Ultramax ships and their performances are compared.

## 3. Data and methodology

The section presents data using for this study, a brief overview of ML methods and model comparison criteria.

### 3.1 Data

**Fig 1:**
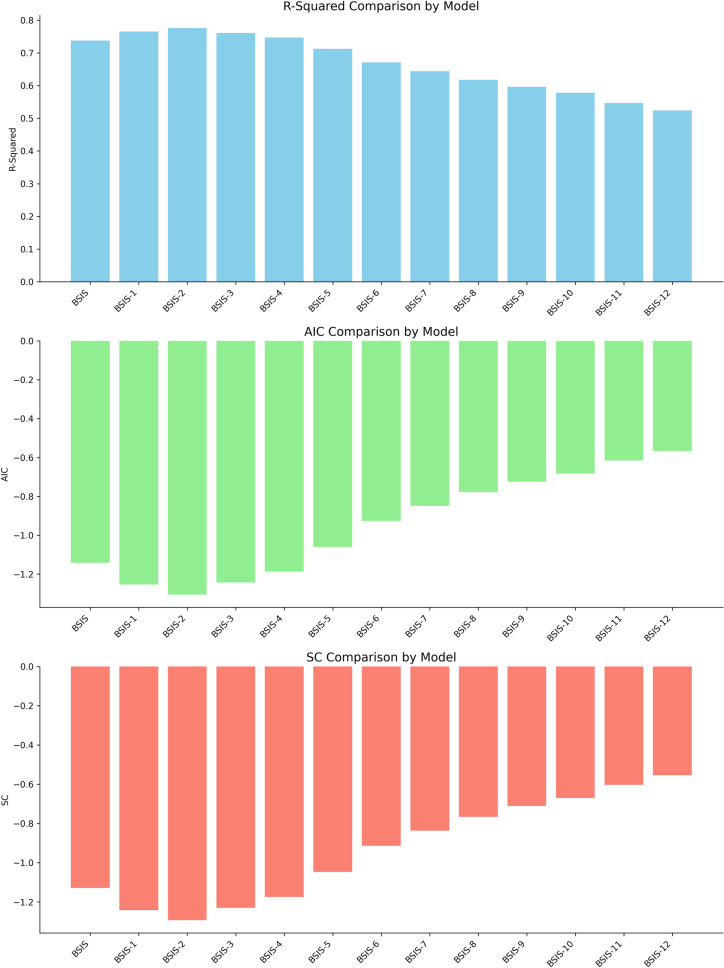
R-squared, AIC and SC comparison for BSIS values.

It is possible to generally divide the variables in the model into market indicator variables and ship factor variables [55]. In the literature, the 1-year time charter rate is widely used as a market indicator. However, there are also researchers in the literature who use Baltic Exchange indices, such as Albertijn et al. [11] and Junior et al. [56]. Since the study is for supramax/ultramax ships, the Baltic Supramax Index (BSIS) is used as the main market indicator in the proposed model. Since the Baltic Supramax Index is used, the time period of the data set covers the period between August 2005, which is the first reachable value of the Baltic Supramax Index, and December 2023. 1-year time charter rates and Indexes are usually handled according to the time of sale. Based on the idea that index values may be reflected in ship prices a few months later; a small preliminary model was established based on the model established by Adland et al. [23] where age, DWT, and freight market (BSIS) are independent variables and ship prices are dependent variables. In the model, all values starting from the BSIS of the month the ship was sold to the BSIS of 12 months before the month the ship was sold were added to the model one by one and the obtained R-squared, Akaike information criteria (AIC), and Schwarz information criteria (SC) values were compared (Fig 1). It is clearly seen in the mini model that the R-squared value of the model including BSIS 2 months before the month of sale is higher than the month of sale, while AIC and SC values are lower. R-squared, AIC and SC values show that the model is much better than other models. The results reinforce the idea that the change in the market is reflected in ship prices a few months later, not in the month of change. This situation may be due to the market participants needing time to reflect the fluctuations in the indexes to the prices and to analyze the changes; and the market reacting to the index values with a delay. In line with the results obtained from the created mini models, two basic models were established in the study. In the first model, the Baltic Exchange indices of the month in which the sale was made were used as freight market indicators; in the second model, in addition to the Baltic Exchange indices of the month in which the sale was made, the Baltic Exchange indices of the months before the sale were also used as freight market indicators. Since the monthly data were used in the study, the index values that included the period of 12 months before the sale were made were taken into consideration. For example, if the ship was sold in April 2023, the independent variable is the value of BSIS in April 2023 (BSIS), its value in March 2023 (BSIS_1), its value in February 2023 (BSIS_2) and its value in January 2023 (BSIS_3) were added to the model. Although the Baltic Supramax Index is used as the basis of the model with the idea that it can better reflect the Supramax/Ultramax secondhand market, other indexes used in the dry bulk cargo market, namely Baltic Capesize Index (BACI), Baltic Panamax Index (BPNI), Baltic Handysize Index (BHSI) was also added to the model as a variable with the idea that they might affect Supramax/Ultramax prices [60–63]. As in BSIS, the values of BACI, BPNI and BHSI covering the 12-month period before the date of sale were taken into account. With regression analysis, only one value was used for each index variable to avoid econometric problems during the determination of variables. For example, the value of a sales of April 2023 for BSIS was either February 2023 or January 2023; both were not included in the model at the same time. In the study, variance inflation factor (VIF) values are especially examined in each model in order to avoid being affected by the multicollinearity problem, which is one of the biggest structural problems that may arise by including the BSIS, BACI, BPNI and BHSI in the regression analysis together. Another variable used as a market indicator is LIBOR, which expresses the cost of capital. The 3-month US Dollar LIBOR value was used in the model [64].

While all ship factor variables and ship sales data until December 2021 are arranged according to the information on the IHS website [65]; Sales values for 2022 and 2023 were obtained from the website of CW Kellock and Co ship brokers. While creating the sample, sales of ships sold before they were put into service, en bloc sales, court sales, and auction sales were not included in the sample as they were thought to be misleading. In addition, the age of the ships that were given the value 0 in the year calculation was taken as 1 in order to avoid any problems when performing the regression analysis. According to IHS, supramax and ultramax ships are classified together, and in this class there are ships in the range of 50 000–67 999 DWT. While obtaining sales data from CW Kellock and Co ship brokers’ website for 2022 and 2023, both this range and its compatibility with the available ship information were taken into account.

In the model, DWT, age, main engine rpm, fei, ship’s crane handling capacity, ship’s total main engine horsepower, builder country (Japan, China, Philippines, South Korea and others), ship main engine manufacturer (MAN - B&W, Wartsila, Mitsubishi, Caterpillar), P&I clubs (Japan P&I Club, Gard, West of England, North of England, UK P&I, Britannia P&I, London P&I Club, Skuld, Steamship Mutual and others), ship’s class (Nippon Kaiji Kyokai, Lloyd’s Register, Bureau Veritas, Det Norske Veritas, American Bureau of Shipping and others) and the ship’s flag (Marshall Islands, Liberia, Malta, Hong Kong, China, Singapore and others) are used as ship factor variables. Among these variables, P&I club and flag variables are not included in the literature as independent variables, and for the first time, the significance of their effects on the ship price was questioned in this study. While the crane handling capacity of the ship is calculated by taking into account the number of cranes on the ship and the crane capacity of each crane, fei is calculated with the formula daily fuel consumption * 1,000,000/ (24 * speed * DWT). In addition, the age of the ship was calculated by subtracting the year the ship was built from the year the ship was sold.

Since ship price, which is the dependent variable, does not comply with normal distribution, ship prices were added to the model by taking its logarithm. Two different data sets were used to create the final model. Using regression analysis with the first data set, it was first investigated whether the variables in the literature were also significant for supramax/ultramax ship prices; then, the determined variables were simplified, and regression analysis was performed again. With the second data set, the performance of the created simplified models on the extended data set was examined. Descriptive statistics of the data set between August 2005 and December 2022 used in the first and second stages are in Table 1 and Table 2; The descriptive statistics of the data set between August 2005 and December 2023 used in the third stage are given in Table 3 and Table 4.

**Table 1:** Descriptive Statistics for Numerical Values (August 2005 – December 2022).

Variable	Minimum	Average	Maximum	Number of Observation
*SecondhandPrice*	2100000	16478776.39	85000000	1819
*SecondhandPrice(Log)*	6.322	7.135	7.929	1819
*DWT*	50029	56698.120	67670	1819
*Age (Year)*	1	10.513	34	1819
*Engines RPM*	75	117.600	136	1796
*FEI*	1.126	1.669	3.094	1785
*FEI(Log)*	0.051	0.219	0.491	1785
*Crane Tone*	43.5	125.220	300	1640
*Total HP Main Eng*	8810	12159.086	18501	1819
*BACI*	-328	2901.109	18749	1819
*BACI_1*	-328	2875.682	18749	1819
*BACI_2*	-328	2737.515	18749	1819
*BACI_3*	-328	2698.371	15357	1819
*BACI_4*	-328	2619.471	18749	1819
*BACI_5*	-328	2673.294	15357	1819
*BACI_6*	-328	2703.192	18749	1819
*BACI_7*	-328	2760.404	18749	1819
*BACI_8*	-328	2803.322	18749	1819
*BACI_9*	-328	2740.672	18749	1819
*BACI_10*	-328	2715.465	18749	1819
*BACI_11*	-328	2788.755	18749	1819
*BACI_12*	-328	2849.855	18749	1819
*BPNI*	287	2031.043	11515	1819
*BPNI_1*	287	2000.560	11515	1819
*BPNI_2*	287	1940.344	11515	1819
*BPNI_3*	287	1903.765	11515	1819
*BPNI_4*	287	1866.317	11515	1819
*BPNI_5*	287	1853.761	11515	1819
*BPNI_6*	287	1877.776	11515	1819
*BPNI_7*	287	1895.334	11515	1819
*BPNI_8*	287	1863.974	11515	1819
*BPNI_9*	287	1861.517	11515	1819
*BPNI_10*	287	1845.913	9967	1819
*BPNI_11*	287	1864.365	11515	1819
*BPNI_12*	287	1872.527	9967	1819
*BSIS*	304	1533.676	6949	1819
*BSIS_1*	304	1517.707	6949	1815
*BSIS_2*	304	1479.690	6949	1809
*BSIS_3*	304	1438.415	6949	1803
*BSIS_4*	304	1417.056	6949	1799
*BSIS_5*	304	1391.688	6949	1794
*BSIS_6*	304	1389.173	6949	1791
*BSIS_7*	304	1395.173	6949	1782
*BSIS_8*	304	1373.112	6949	1777
*BSIS_9*	304	1362.624	6949	1771
*BSIS_10*	304	1350.177	6405	1762
*BSIS_11*	304	1344.728	6949	1755
*BSIS_12*	304	1336.795	6405	1749
*BHSI*	214	843.617	3304	1761
*BHSI_1*	214	833.785	3304	1755
*BHSI_2*	214	812.736	3304	1749
*BHSI_3*	214	789.821	3198	1744
*BHSI_4*	214	775.159	3304	1738
*BHSI_5*	214	763.370	3198	1733
*BHSI_6*	214	765.020	3304	1728
*BHSI_7*	214	764.921	3304	1725
*BHSI_8*	214	750.848	3304	1712
*BHSI_9*	214	746.629	3304	1703
*BHSI_10*	214	734.514	3304	1690
*BHSI_11*	214	730.590	3304	1686
*BHSI_12*	214	725.739	3304	1680
*LIBOR*	0.001227	0.0149	0.055079	1819

**Table 2:** Distribution of Dummy Variables (August 2005 – December 2022).

Flag	Rate	Class	Rate		
**Panama**	29.5	Nippon Kaiji Kyokai (IACS)	40.4		
**Marshall Islands**	15.6	Lloyd’s Register (IACS)	14.8		
**Liberia**	9.1	Bureau Veritas (IACS)	13		
**Malta**	6.8	Det Norske Veritas (IACS)	9.5		
**Hong Kong, China**	8.1	American Bureau of Shipping (IACS)	9.3		
**Singapore**	7	Others	11.2		
**Others**	19.9	With Two Classes	1.5		
**With two flags**	4	Disclassed	0.3		
**P&I Club**	**Rate**	**Main Engine Manufacturer**	**Rate**	**Builder Country**	**Rate**
**Japan P&I Club**	13.4	MAN-B&W	87.3	Japan	49.4
**Gard**	12.3	Wartsila	9.1	China	33.7
**West of England**	11.1	Mitsubishi	3.5	Philippines	7.1
**North of England**	10	Caterpillar	0.1	Korea, South	4.1
**UK P&I**	9.1			Others	5.7
**Britannia P&I**	8				
**London P&I Club**	7.5				
**Skuld**	6.4				
**Steamship Mutual**	4.8				
**Others**	13.4				
**Unknown**	4				

**Table 3:** Descriptive Statistics for Numerical Values (August 2005 – December 2023).

Variable	Minimum	Average	Maximum	Number of Observation
*Secondhandprice*	2100000	16582815	85000000	2005
*SecondhandPrice(Log)*	6.322	7.143	7.929	2005
*DWT*	50029	56823.34	67670	2005
*Age(Year)*	1	10.618	34	2005
*BACI*	-328	2829.498	18749	2005
*BACI_2*	-328	2628.722	18749	2005
*BSIS*	304	1489.783	6949	2005
*BSIS_2*	304	1435.225	6949	1995
*LIBOR*	0.001227	0.018521	0.056615	2005

**Table 4:** Distribution of Dummy Variables (August 2005 – December 2023).

Builder Country	Rate
*Japan*	48.33
*China*	34.96
*Others*	16.71

As can be clearly seen in Table 1, the number of observations is not the same for each variable. Since regression analysis cannot be performed with missing observations, the regression analyses created vary depending on the number of observations of the variables. In particular, the Crane tone variable, which expresses the crane handling capacity of the ship, causes serious losses in the data set. Since the data for the Baltic Handysize Index starts from June 2006, this index also affects the sample size.

While the majority of the ships sold between August 2005 and December 2022 were operated under flag of convenience; The top three are the Panama flag with 30%, the Marshall Islands flag with 16%, and the Liberia flag with 9%. Among the class organizations the ships are affiliated with, Nippon Kaiji Kyokai ranks first with 40%. Lloyd’s Register follows it with 15% and Bureau Veritas with 13%. Although there is no club with a particularly dominant proportion of P&I clubs, the top three are Japan P&I Club (13%), Gard (12%), and West of England (11%). Among machinery manufacturing companies, MAN-B&W is present at a rate of 87%. When ship construction countries are examined, it is seen that Japan ranks first with a rate of 49%, China ranks second with a rate of 34%, and the Philippines ranks third with a rate of 7% (Table 2).

For August 2005 – December 2023, there was no change in the largest and smallest values of any variable except the largest value of the LIBOR variable. However, the mean value of all variables has changed. While the number of samples increased from 1819 to 2005 for the dependent variable; The second delay of BSIS reduces the sample number the most (Table 3).

Only the shipbuilding country is included as a dummy variable between August 2005 and December 2023. While 48% of the ships in the sample were built in Japan, 35% were built in China (Table 4).

### 3.2 Methods

In the study, predictions were made using Linear Regression, Decision Tree, Random Forest and XGBoost ML algorithms on 2 models created with linear regression. The 2 models created and the predictions made based on these models were compared using R^2^, RMSE, MAE, and MAPE values. While the predictions were made via the Python program, the performance values of the models were calculated in Excel. The Table 5 shows the process from uploading the data into Python to calculating the prediction results. While the process works the same for all models, it differs in the creation and training of the models in section 4. The parameters used especially for the Decision Tree, Random Forest and XGBoost models are specified in the Table 5. Another point that can be noted in the algorithm is that the X variables change according to the established models. The process is repeated in the same way according to the SPlog and x variables uploaded into Python.

**Table 5:** The algorithm of the applied models.

**1. Input** * Dataset* Independent variables (X) Dependent variable (Y) * Target variable* y=SPLog * Features* All variables except SPLog
**2. Data** * Upload the Dataset* dataFrame = pd.read_excel(“DATAINPUT.xlsx”) * Clean missing values* dataFrame = dataFrame.dropna() * Separate target (y) and independent variables (X)* y = dataFrame[“SPLog”].values X = dataFrame.drop(“SPLog”, axis=1).values
**3. Split data into Training and Test Set** * Split data* x_train, x_test, y_train, y_test = train_test_split(X, y, test_size=0.3, random_state=42) * Standardize data* sc = StandardScaler() x_train = sc.fit_transform(x_train) x_test = sc.transform(x_test)
**4. Build and Train Models**
**4.1. Linear Regression** * Define the model* lin_model = LinearRegression() * Train the model* lin_model.fit(x_train, y_train) * Make prediction on test data* lin_pred = lin_model.predict(x_test)
**4.2. Decision Tree** * Define the model* dt_model = DecisionTreeRegressor(random_state=0, max_depth=12, max_features=6, min_samples_leaf=5, min_samples_split=2) * Train the model* dt_model.fit(x_train, y_train) * Make prediction on test data* dt_pred = dt_model.predict(x_test)
**4.3. Random Forest** * Define the model* rf_model = RandomForestRegressor(random_state=0, max_depth=12, max_features=3, min_samples_leaf=1, min_samples_split=2, n_estimators=100) * Train the model* rf_model.fit(x_train, y_train) * Make prediction on test data* rf_pred = rf_model.predict(x_test)
**4.4. XGBoost** * Define the model* xgb_model = XGBRegressor(alpha=0, colsample_bytree=0.9, gamma=0, reg_lambda=1, learning_rate=0.1, max_depth=6, n_estimators=200, subsample=0.9) * Train the model* xgb_model.fit(x_train, y_train) * Make prediction on test data* xgb_pred = xgb_model.predict(x_test)
**5. Combine Prediction Results** * Add prediction results to a list* predictions = [(y_test), (lin_pred), (dt_pred), (rf_pred), (xgb_pred)] * Convert the list to a DataFrame and save* output = pd.DataFrame(predictions) output.to_excel(“DATAOUTPUT.xlsx”) **6. Measure the performance of models** * Obtain real values of logarithmic prediction results Parametres of performance in Excel* * Obtaining R*^*2*^*, RMSE, MAE, MAPE values from real values in Excel*

#### 3.2.1. *Linear regression.*

Linear regression, the simplest and classical model among regression models, or ordinary least squares; finds the “slope” ($\beta_{j}$) and constant ($\beta_{0}$) parameters, called the weight or coefficient, that minimize the mean square error between the actual value of the variable to be predicted and the value predicted by the model [31]. Linear regression;


$$f\left(X\right)=\;\beta_{0}+\;\underset{j=1}{\overset{p}{\sum }}\;X_{j}\beta_{j}$$

is expressed in the form. In the linear model, the regression function assumes that E(Y|X) is linear or that the linear approach is logical in the model to be established. $X_{j}$ ‘s (independent, explanatory variable) in linear regression could be: quantitative inputs; transformed forms of quantitative inputs by logarithm, square root or square, etc.; Again, polynomial forms of quantitative inputs such as $X_{1}^{3}$; numerical values where qualitative data is converted into quantitative data by coding; and interactions between variables such as $X_{3}=\;X_{1}*X_{2}$. Obtaining $X_{j}$ in different ways does not affect the linearity of the model in terms of parameters [66].

In regression models, the least squares method produces a t statistic for each parameter and a p value expressing the significance of this statistic. While the $H_{0}$ hypothesis of the t statistics of the variables states that the coefficient of the variable is equal to 0; $H_{1}$ hypothesis states that the coefficient of the variable is different from 0. Therefore, as the p values of the coefficient numbers of the variables decrease, the $H_{1}$ hypothesis cannot be rejected, that is, the coefficient of the variable becomes significant for the model. When evaluating the hypothesis, the p value can be evaluated according to 0.1, 0.05 and 0.01. It is the decision of the researcher according to which value the hypothesis will be evaluated. When interpreting the p value of the coefficient, using the value 0.05 to reject the hypothesis indicates that if the p value is 0.06, the $H_{0}$ hypothesis cannot be rejected, that is, the coefficient is equal to 0 [67].

The primary purpose of using Linear Regression in the study is to determine the independent variables for the models to be used in ship valuation. In line with this purpose, in the first stage of the applied methodology, regression models were created based on the basic model where the logarithm of ship prices is the dependent variable and the ship’s age, dwt and Baltic Exchange index are the independent variables. First of all, models were created that take into account the Baltic Exchange index values in the month the ship was sold as a market indicator. The regression equations of the best five models created by considering the Baltic Exchange index values in the month the ship was sold in Table 6 are as follows:

**Table 6:** The best 5 models, created by taking into account the Baltic Exchange indices values in the month the ship was sold.

*Model Rank*	*Independent Variables*	*R* ^*2*^	*AIC*	*SC*
1.	Age, DWT, LIBOR, BACI, BSIS, Japan, China, UK P&I, Skuld, American Bureau of Shipping, Caterpillar, Engine RPM, Total HP Main Engine	0.792732	-1.376329	-1.333508
2.	Age, DWT, LIBOR, BSIS, Japan, China, UK P&I, Skuld, American Bureau of Shipping, Caterpillar, Engine RPM, Total HP Main Engine	0.790600	-1.367208	-1.327446
3.	Age, DWT, LIBOR, BHSI, Japan, China, UK P&I, Skuld, American Bureau of Shipping, Caterpillar, Engine RPM, Total HP Main Engine	0.781256	-1.339038	-1.298290
4.	Age, DWT, LIBOR, BPNI, Japan, China, American Bureau of Shipping, Caterpillar, Engine RPM, Total HP Main Engine	0.757174	-1.221338	-1.187693
5.	Age, DWT, LIBOR, BACI, Japan, China, UK P&I, Skuld, Caterpillar, Engine RPM, Total HP Main Engine	0.703334	-1.019963	-0.983259

(1)
$\text{ln}\left(SP\right)=\beta_{0}+\beta_{1}AGE+\;\beta_{2}DWT+\;\beta_{3}LIBOR+\;\beta_{4}BACI+\;\beta_{5}BSIS+\;\beta_{6}Japan+\;\beta_{7}China+\;\beta_{8}UKP\&I+\beta_{9}Skuld+\;\beta_{10}ABS+\;\beta_{11}Caterpillar+\;\beta_{12}RPM+\;\beta_{13}HP+\;\varepsilon$

(2)
$\begin{array}{c} \text{ln}\left(SP\right)\;=\;\beta_{0}+\beta_{1}AGE+\;\beta_{2}DWT+\;\beta_{3}LIBOR+\;\beta_{4}BSIS+\;\beta_{5}Japan+\;\beta_{6}China+\;\beta_{7}UKP\&I+\;\beta_{8}Skuld\;\\ +\;\beta_{9}ABS+\;\beta_{10}Caterpillar+\;\beta_{11}RPM+\;\beta_{12}HP+\;\varepsilon \end{array}$

(3)
$\begin{array}{c} \text{ln}\left(SP\right)\;=\;\beta_{0}+\beta_{1}AGE+\;\beta_{2}DWT+\;\beta_{3}LIBOR+\;\beta_{4}BHSI+\;\beta_{5}Japan+\;\beta_{6}China+\;\beta_{7}UKP\&I+\;\beta_{8}Skuld\\ +\;\beta_{9}ABS+\;\beta_{10}Caterpillar+\;\beta_{11}RPM+\;\beta_{12}HP+\;\varepsilon \end{array}$

(4)
$\begin{array}{c} \text{ln}\left(SP\right)\;=\;\beta_{0}+\beta_{1}AGE+\;\beta_{2}DWT+\;\beta_{3}LIBOR+\;\beta_{4}BPNI+\;\beta_{5}Japan+\;\beta_{6}China+\;\beta_{7}ABS+\;\beta_{8}Caterpillar\\ +\;\beta_{9}RPM+\;\beta_{10}HP+\;\varepsilon \end{array}$

(5)
$\begin{array}{c} \text{ln}\left(SP\right)\;=\;\beta_{0}+\beta_{1}AGE+\;\beta_{2}DWT+\;\beta_{3}LIBOR+\;\beta_{4}BACI+\;\beta_{5}Japan+\;\beta_{6}China+\;\beta_{7}UKP\&I\\ +\;\beta_{8}Skuld+\;\beta_{9}ABS+\;\beta_{10}Caterpillar+\;\beta_{11}RPM+\;\beta_{12}HP+\;\varepsilon \end{array}$

In the regression equations created, ln(SP) represents the logarithm of the ship prices, while $\beta_{0}$ is the constant term, $\beta_{1}$, $\beta_{2}$,…, $\beta_{13}$ represents the coefficients of each independent variable and ε represents the error term. The independent variables in these equations are as follows:

Ship age (AGE), ship carrying capacity (DWT), 3-month LIBOR value (LIBOR), Baltic Capesize Index (BACI), Baltic Supramax Index (BSIS), Baltic Handysize Index (BHSI), Baltic Panamax Index (BPNI), Japan as a shipbuilding country (Japan), China as a shipbuilding country (China), UK P&I Club (UKP&I), Skuld (Skuld), American Ship Classification Society (ABS), Caterpillar (Caterpillar), main engine rpm (RPM) and ship’s total main engine horsepower (HP).

Then, as a market indicator, models were created that took into account the Baltic Exchange index values in the month of sale of the ship and the months before the month of sale. The regression equations of the best five models created by taking into account the Baltic Exchange index values in the month of sale of the ship and the months before the month of sale in Table 7 are as follows, according to the ranking in Table 7:

**Table 7:** The best 5 models created by considering the values of the Baltic Exchange indices in the month of sale and in the past months.

*Model Rank*	*Independent Variables*	*R* ^*2*^	*AIC*	*SC*
1.	Age, DWT, LIBOR, BACI_2, BSIS_2, Japan, China, UK P&I, American Bureau of Shipping, Caterpillar, Engine RPM	0.825909	-1.558346	-1.521475
2.	Age, DWT, LIBOR, BSIS_2, Japan, China, UK P&I, American Bureau of Shipping, Caterpillar, Engine RPM	0.825226	-1.555551	-1.521752
3.	Age, DWT, LIBOR, BSIS_1, BACI_1, BPNI_3, Japan, China, UK P&I, American Bureau of Shipping, Caterpillar, Engine RPM	0.824981	-1.547396	-1.507561
4.	Age, DWT, LIBOR, BSIS_3, BACI_3, BPNI_1, Japan, China, UK P&I, Caterpillar, Engine RPM	0.823406	-1.547210	-1.510254
5.	Age, DWT, LIBOR, BSIS_1, BPNI_3, Japan, China, UK P&I, Caterpillar, Engine RPM	0.821043	-1.527381	-1.493674

(1)
$\begin{array}{c} \text{ln}\left(SP\right)=\;\beta_{0}+\beta_{1}AGE+\;\beta_{2}DWT+\;\beta_{3}LIBOR+\;\beta_{4}BACI\_2+\;\beta_{5}BSIS\_2+\;\beta_{6}Japan+\;\beta_{7}China\\ +\;\beta_{8}UKP\&I+\;\beta_{9}ABS+\;\beta_{10}Caterpillar+\;\beta_{11}RPM+\;\varepsilon \end{array}$

(2)
$\begin{array}{c} \text{ln}\left(SP\right)=\;\beta_{0}+\beta_{1}AGE+\;\beta_{2}DWT+\;\beta_{3}LIBOR+\;\beta_{4}BSIS\_2+\;\beta_{5}Japan+\;\beta_{6}China+\;\beta_{7}UKP\&I\\ +\;\beta_{8}ABS+\;\beta_{9}Caterpillar+\;\beta_{10}RPM+\;\varepsilon \end{array}$

(3)
$\begin{array}{c} \;\;\;\;\;\;\text{ ln}\left(SP\right)=\;\beta_{0}+\beta_{1}AGE+\;\beta_{2}DWT+\;\beta_{3}LIBOR+\;\beta_{4}BSIS\_1+\;\beta_{5}BACI\_1+\;\beta_{6}BPNI\_3+\;\beta_{7}Japan+\;\beta_{8}China\\ +\;\beta_{9}UKP\&I+\;\beta_{10}ABS+\;\beta_{11}Caterpillar+\;\beta_{12}RPM+\;\varepsilon \end{array}$

(4)
$\begin{array}{c} \text{ln}\left(SP\right)=\;\beta_{0}+\beta_{1}AGE+\;\beta_{2}DWT+\;\beta_{3}LIBOR+\;\beta_{4}BSIS\_3+\;\beta_{5}BACI\_3+\;\beta_{6}BPNI\_1+\;\beta_{7}Japan+\;\beta_{8}China\\ +\;\beta_{9}UKP\&I+\;\beta_{10}Caterpillar+\;\beta_{11}RPM+\;\varepsilon \end{array}$

(5)
$\begin{array}{c} \text{ln}\left(SP\right)=\;\beta_{0}+\beta_{1}AGE+\;\beta_{2}DWT+\;\beta_{3}LIBOR+\;\beta_{4}BSIS\_1+\;\beta_{5}BPNI\_3+\;\beta_{6}Japan+\;\beta_{7}China\\ +\;\beta_{8}UKP\&I+\;\beta_{9}Caterpillar+\;\beta_{10}RPM+\;\varepsilon \end{array}$

In the regression equations created, ln(SP) represents the logarithm of the ship prices, while $\beta_{0}$ is the constant term, $\beta_{1}$, $\beta_{2}$,…, $\beta_{13}$ represents the coefficients of each independent variable and ε represents the error term. The independent variables in these equations are as follows:

Ship age (AGE), ship carrying capacity, Deadweight tonnage (DWT), 3-month LIBOR value (LIBOR), Baltic Capesize Index one month before the ship sale (BACI_1), Baltic Capesize Index two months before the ship sale (BACI_2), Baltic Capesize Index three months before the ship sale (BACI_3), Baltic Supramax Index one month before the ship sale (BSIS_1), Baltic Supramax Index two months before the ship sale (BSIS_2), Baltic Supramax Index three months before the ship sale (BSIS_3), Baltic Panamax Index one month before the ship sale (BPNI_1), Baltic Panamax Index three months before the ship sale (BPNI_3), Japan as a shipbuilding country (Japan), China as a shipbuilding country (China), UK P&I Club (UKP&I), American Bureau of Shipping (ABS), Caterpillar (Caterpillar) and main engine rpm (RPM).

The two models that were ranked best in Table 6 and Table 7 and whose regression equations are given below were the models used in the comparison in the rest of the study.


$$\left(\text{M}1\right)\;\;\;\;\;\;\text{ ln}\left(SP\right)\;=\;\beta_{0}+\beta_{1}AGE+\;\beta_{2}DWT+\;\beta_{3}LIBOR+\;\beta_{4}BACI+\;\beta_{5}BSIS+\;\beta_{6}Japan+\;\beta_{7}China+\;\beta_{8}UKP\&I+\;\beta_{9}Skuld+\;\beta_{10}ABS+\;\beta_{11}Caterpillar+\;\beta_{12}RPM+\;\beta_{13}HP+\;\varepsilon$$

$$\left(\text{M}2\right)\;\;\;\;\;\;\text{ ln}\left(SP\right)=\;\beta_{0}+\beta_{1}AGE+\;\beta_{2}DWT+\;\beta_{3}LIBOR+\;\beta_{4}BACI\_2+\;\beta_{5}BSIS\_2+\;\beta_{6}Japan+\;\beta_{7}China+\;\beta_{8}UKP\&I+\;\beta_{9}ABS+\;\beta_{10}Caterpillar+\;\beta_{11}RPM+\;\varepsilon$$

In the regression equations created, ln(SP) represents the logarithm of the ship prices, while $\beta_{0}$ is the constant term, $\beta_{1}$, $\beta_{2}$,…, $\beta_{13}$ represents the coefficients of each independent variable and ε represents the error term. The independent variables in these equations are as follows:

Ship age (AGE), ship carrying capacity (DWT), 3-month LIBOR value (LIBOR), Baltic Capesize Index (BACI), Baltic Capesize Index two months before the ship sale (BACI_2), Baltic Supramax Index (BSIS), Baltic Supramax Index two months before the ship sale (BSIS_2), Japan as a shipbuilding country (Japan), China as a shipbuilding country (China), UK P&I Club (UKP&I), Skuld (Skuld), American Ship Classification Society (ABS), Caterpillar (Caterpillar), main engine rpm (RPM) and ship’s total main engine horsepower (HP).

In the third stage of the study, the two models compared were simplified. The regression equations of the simplified models are as follows:


$$\left(\text{M}1\right)\;\;\;\;\;\;\text{ ln}\left(SP\right)\;=\;\beta_{0}+\beta_{1}AGE+\;\beta_{2}DWT+\;\beta_{3}LIBOR+\;\beta_{4}BACI+\;\beta_{5}BSIS+\;\beta_{6}Japan+\;\beta_{7}China+\varepsilon$$

$$\left(\text{M}2\right)\;\;\;\;\;\;\text{ ln}\left(SP\right)=\;\beta_{0}+\beta_{1}AGE+\;\beta_{2}DWT+\;\beta_{3}LIBOR+\;\beta_{4}BACI\_2+\;\beta_{5}BSIS\_2+\;\beta_{6}Japan+\;\beta_{7}China+\;\varepsilon$$

In the regression equations created, ln(SP) represents the logarithm of the ship prices, while $\beta_{0}$ is the constant term, $\beta_{1}$, $\beta_{2}$,…, $\beta_{13}$ represents the coefficients of each independent variable and ε represents the error term. The independent variables in these equations are as follows:

Ship age (AGE), ship carrying capacity (DWT), 3-month LIBOR value (LIBOR), Baltic Capesize Index (BACI), Baltic Capesize Index two months before the ship sale (BACI_2), Baltic Supramax Index (BSIS), Baltic Supramax Index two months before the ship sale (BSIS_2), Japan as a shipbuilding country (Japan) and China as a shipbuilding country (China).

#### 3.2.2. *Decision tree/regression tree.*

The decision tree model, which can be used in both classification and regression problems, is a ML algorithm consisting of nodes, branches and leaves. A decision tree that starts with a root node progresses from the root node to decision nodes and finally to terminal nodes that express the final value for that route [68].

In decision trees created to solve the regression problem; When dividing the node into sub-nodes, the maximum reduction in variance is taken into account as the split criterion, and nodes are divided according to this criterion unless a stopping criterion comes into play. When applying the decision tree algorithm, first (1) start with all observations, that is, a single node, and take the mean and variance of the target variable; (2) The decrease in variance is calculated for each variable, taking into account its potential to become the next node, and the variable that reduces the variance the most is selected as the node. (3) For each leaf node; It stops if the number of observations in a node is less than the threshold value or if the maximum variance reduction of any of the variables is less than a certain threshold value. If it is not less, the 2nd stage is repeated [68].

In addition to being an algorithm that is easy to understand and apply, decision trees do not require much data cleaning because they are not affected by outliers and missing values [68]. In addition, preliminary processes such as normalization or standardization are not required in decision trees [31]. Decision trees come to the fore especially in data sets where it is difficult to build a model with a linear regression model and where variables interact in complex and non-linear ways. Decision trees are also very useful in selecting important variables for prediction [68]. The biggest disadvantage of decision trees is that they tend to provide overfitting and poor generalization performance [31].

#### 3.2.3. *Random forest.*

Decision trees form the basis of random forest algorithms [32]. The random forest algorithm, developed by Leo Breiman and Adele Cutler [69], is a predictive algorithm within the scope of ensemble learning algorithms [68]. The main logic in community learning is; rather than the solution of an estimator who can produce the best solution to the problem in a community; The collective solution of the estimators in the community will be better than the solution of a single estimator [32]. With this logic, ensemble learning algorithms consist of the combination of various independent models, similar or different, to solve a prediction model [68]. Bootstrap aggregating, boosting and stacking are the most used ensemble learning algorithms [32]. Similar independent models are created in the bootstrap aggregating method. What is meant by similar, independent models; Take decision trees, for example; It is the case that there are trees with different depths and trees with only some variables. The average of all models used in prediction gives the final result for the solution of the problem. In the boosting method, weak models are used, each model is gradually improved by correcting the errors of the previous models, and strong models are obtained [68]. Random forests appear as a solution method that uses the bootstrap aggregating method from ensemble learning algorithms to solve the overfitting problem, which is the biggest disadvantage of decision trees. Random forests reduce overfitting by creating decision trees, each of which works well and overfits in different ways, and by averaging the created decision trees [31].

In random forest algorithms, randomness occurs by selecting samples or features. Boostrap sampling is used when creating trees in random forest algorithms. In Bosstrap sampling, the same data can be used repeatedly in selected samples to create models. Since the selected sample size must be the same as the main sample size, some data are repeated and some data are removed from the sample. For example, when choosing a sample from a set containing [“a”, “b”, “c”, “d”], the selected sample can be [“a”, “b”, “b”, “d”]. In the selected sample, the letter “c” is not used while “b” is used more than once. The randomness of features includes the logic of using subsets selected from all features, that is, different features are used in each node. These two situations ensure that all trees in the random forest are different from each other, which increases the overall performance of the model [31].

In random forest algorithms, scaling is not done as in decision trees. While random forest algorithms share all the benefits of decision trees, they also address some of their shortcomings. The reason decision trees are still used is because there is a need for a compact representation of the decision-making process. Random forest algorithms take more time to train and predict than linear models. If time and memory are important in the application, it may be more appropriate to choose a linear model instead of random forest algorithms [31].

#### 3.2.4. *XGBoost.*

With the acceleration in big data, studies on ML algorithms that will produce optimum predictions with big data have also begun. Although decision trees, one of these studies, showed high compatibility with the data and obtained accurate results, they were incomplete in generalizing the models created to new data. However, ensemble methods combining bagging and boosting can provide solutions to the generalization problems of decision trees. The consistency, power, and superior results of gradient boosting, one of the prominent algorithms, pushed Tianqi Chen to work on this algorithm and improved the algorithm by making additions to gradient boosting [70]. XGBoost, one of the dominant ML algorithms for classification and regression problems, was published by Tianqi Chen as a research project in 2014 [71]. Details of the XGBoost method are included in the article published by Chen et al. [72].

The general principles of gradient boosting are used in the model built on the basis of decision trees [71]. Gradient boosting creates new models that predict the errors or residuals of previous models and makes the final prediction by combining these models. Gradient boosting, which can be used in both regression and classification problems, uses a gradient descent algorithm to minimize loss when adding new models [73]. One of the features that makes XGBoost faster, more accurate and more preferable than other methods; When faced with missing data, it scores different splitting options and chooses the one with the best results. Another is the features included in the design of XGBoost to provide a speed advantage. These features are approximate split-finding algorithm, sparsity aware split-finding, parallel computing, cache-aware access, block compression and sharding [70]. Unlike gradient boosting algorithms, which build trees sequentially, XGBoost builds trees in parallel, which makes the model faster than other tree-based algorithmsXGBoost uses an approximate algorithm to find the best split points. The approximate split method uses separate splittings to combine continuous features, which speeds up model training. XGBoost also includes another tree-growing method that uses a histogram-based algorithm to split continuous features into discrete partitions. XGBoost’s ability to control model complexity and reduce overfitting through regularization is effective in better prediction performance [71]. The purpose of built-in regularization is to add information to reduce variance and prevent overfitting. In XGBoost, which is the regularized version of gradient boosting; while regulated parameters penalize complexity; smoothes the final weights to prevent overfitting [70].

### 3.3. *Performance comparision of models*

In the study, $R^{2}$, RMSE, MAE, MAPE values were used as performance analysis criteria when comparing models.


$${\text{R}}^{2}=1-\;\frac{\sum_{i=1}^{n}{\left(y_{i}-\hat{y_{i}}\right)}^{2}}{\sum_{i=1}^{n}{\left(y_{i}-\bar{y}\right)}^{2}}$$

$$\text{Root Mean Squared Error }\left(\text{RMSE}\right)\;=\sqrt{\frac{1}{n}\underset{i=1}{\overset{n}{\sum }}\;{\left(y_{i}-\hat{y_{i}}\right)}^{2}}$$

$$\text{Mean Absolute Error }\left(\text{MAE}\right)\;=\frac{1}{n}\underset{i=1}{\overset{n}{\sum }}\;\left|y_{i}-\hat{y_{i}}\right|$$

$$\text{Mean Absolute Percent Error }\left(\text{MAPE}\right)\;=\frac{1}{n}\underset{i=1}{\overset{n}{\sum }}\;\left|\frac{y_{i}-\hat{y_{i}}}{y_{i}}\right|*100$$

In the formulas of the criteria [67]; $y_{i}$: Actual values, $\hat{y_{i}}$: Predicted values, $\bar{y}$: Average of actual values, n: Number of observations represents.

## 4. Modelling procedure

**Fig 2:**
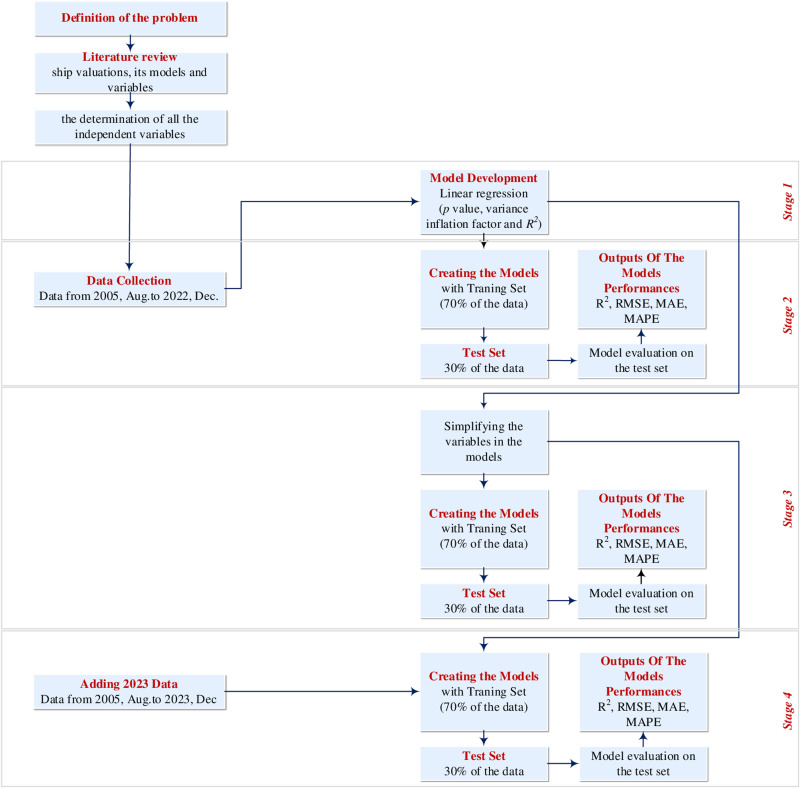
Applied Methodology.

In order to determine whether the independent variables in the study are significant for Supramax/Ultramax ship prices, linear regression models were created in EViews10; and through the best two models, in the Python program; prediction was made using Linear Regression, Decision Tree, Random Forest and XGBoost ML algorithms, and performance analysis values were obtained. The selection of the best model for the prediction of the prices of Supramax/Ultramax ships took place in four stages (Fig 2):

(i) In the first stage, using the entire data set between August 2005 and December 2022, P&I and ship’s flag variables were added to all variables in the literature and accessible to researchers in determining the ship price; linear regression analyses were conducted in which the logarithm of the ship’s sales prices was the dependent variable. While performing linear regression analyses, models were established by taking into account the values of the Baltic Exchange indices, one of the market indicators, in the month the ship was sold, and the best model was determined according to the AIC, SC and R-squared values [67]. Then, models were established based on the values of the Baltic Exchange indices in the month of sale and in the past months, and the best model was determined again according to the AIC, SC and R-squared values. While determining the best models for both cases, attention was paid to ensure that the p value was greater or less than 0.05 and that all conditions that the linear regression model should provide were met.
(ii) After determining the regression models to be used to predict supramax/ultramax ships in the first stage; In the second stage, using the determined regression models, price prediction was made with Linear Regression, Decision Tree, Random Forest and XGBoost ML algorithms. In order to compare the models, 30% of the data set was separated as the test set and 70% as the training set. The coefficients of 2 regression models determined with the 70% training set were determined by Linear Regression, Decision Tree, Random Forest and XGBoost ML methods, and the ship prices in the 30% test data were predicted with the created models. R^2^, RMSE, MAE, and MAPE performance analysis values were used to compare the models.

Broker reports generally include the ship’s sale date, name, type, DWT, build year, build country, number of cranes and tonnage of cranes, special circumstances of the ship, ship sales price, and buyer country. In order to use the models created in the first stage; The appraiser needs to know the ship’s rpm, P&I club and ship machinery manufacturer for the ships in the sample. However, accessing this information may cause both an extra cost and a waste of time for the appraiser.

(iii) In the third stage, variables that cannot be easily accessed in broker reports (*engine rpm, horsepower, P&I club, machine manufacturer, class*) were removed from the regression models created in the first stage, and price predictions were made with the new regression models created. The aim of this stage is to see the effect of the extracted independent variables on prediction.
(iv) In the fourth stage, the results of the simplified models were re-evaluated by adding ship sales data for 2023 to the data set in order to see how the simplified models worked when the data set was expanded and to address the performance of the models.

## 5. Results and discussion

In the first stage; regression models were established where logarithm of secondhand ship prices were the dependent variable, BSIS, BACI, BPNI and BHSI values in the month of sale, LIBOR, DWT, age, main engine rpm, fei, ship’s crane handling capacity, ship’s total main engine horsepower, builder country (Japan, China, Philippines, South Korea and others), ship main engine manufacturer (MAN - B&W, Wartsila, Mitsubishi, Caterpillar), P&I clubs (Japan P&I Club, Gard, West of England, North of England, UK P&I, Britannia P&I, London P&I Club, Skuld, Steamship Mutual and others), ship’s class (Nippon Kaiji Kyokai, Lloyd’s Register, Bureau Veritas, Det Norske Veritas, American Bureau of Shipping and others) and the ship’s flag (Marshall Islands, Liberia, Malta, Hong Kong, China, Singapore and others) were the independent variable options. While creating the models, age, DWT, and freight market (BSIS, BACI, BPNI and BHSI), which are independent variables in almost all studies, were taken as basis and other variables were added one by one. The most important points while adding and removing were that the p values of the variables were less than 0.05 and the VIF values were less than 10. In addition to these two important points, HAC correction was made in order not to be affected by autocorrelation and heteroscedasticity problems. It was assumed that the errors were normally distributed due to the sample size. The models that meet these conditions were ranked according to their R-squared, AIC and SC. The models in the first 5 according to this ranking, are given in Table 6. The model that includes LIBOR, BACI, BSIS as market indicators and age, DWT, shipbuilding country (Japan and China), P&I Club (UK P&I and Skuld), Class (American Bureau of Shipping), ship engine manufacturer (Caterpillar), engines rpm and total hp main engine as the ship’s characteristics is the best model. The model to be used in the comparison in the rest of the study will be called Model 1.

Regression models were established where logarithm of ship prices were the dependent variable, BSIS, BSIS_1, BSIS_2, BSIS_3, BSIS_4, BSIS_5, BSIS_6, BSIS_7, BSIS_8, BSIS_9, BSIS_10, BSIS_11, BSIS_12, BACI, BACI_1, BACI_2, BACI_3, BACI_4, BACI_5, BACI_6, BACI_7, BACI_8, BACI_9, BACI_10, BACI_11, BACI_12, BPNI, BPNI_1, BPNI_2, BPNI_3, BPNI_4, BPNI_5, BPNI_6, BPNI_7, BPNI_8, BPNI_9, BPNI_10, BPNI_11, BPNI_12, BHSI, BHSI_1, BHSI_2, BHSI_3, BHSI_4, BHSI_5, BHSI_6, BHSI_7, BHSI_8, BHSI_9, BHSI_10, BHSI_11, BHSI_12, LIBOR, DWT, age, main engine rpm, fei, ship’s crane handling capacity, ship’s total main engine horsepower, builder country (Japan, China, Philippines, South Korea, and others), ship main engine manufacturer (MAN - B&W, Wartsila, Mitsubishi, Caterpillar), P&I clubs (Japan P&I Club, Gard, West of England, North of England, UK P&I, Britannia P&I, London P&I Club, Skuld, Steamship Mutual and others), ship’s class (Nippon Kaiji Kyokai, Lloyd’s Register, Bureau Veritas, Det Norske Veritas, American Bureau of Shipping and others) and the ship’s flag (Marshall Islands, Liberia, Malta, Hong Kong, China, Singapore and others) were the independent variable options. While creating the models, age, DWT and freight market (BSIS, BSIS_1, BSIS_2, BSIS_3, BSIS_4, BSIS_5, BSIS_6, BSIS_7, BSIS_8, BSIS_9, BSIS_10, BSIS_11, BSIS_12, BACI, BACI_1, BACI_2, BACI_3, BACI_4, BACI_5, BACI_6, BACI_7, BACI_8, BACI_9, BACI_10, BACI_11, BACI_12, BPNI, BPNI_1, BPNI_2, BPNI_3, BPNI_4, BPNI_5, BPNI_6, BPNI_7, BPNI_8, BPNI_9, BPNI_10, BPNI_11, BPNI_12, BHSI, BHSI_1, BHSI_2, BHSI_3, BHSI_4, BHSI_5, BHSI_6, BHSI_7, BHSI_8, BHSI_9, BHSI_10, BHSI_11, BHSI_12), which are independent variables in almost all studies, were taken as basis and other variables were added one by one. The most important points while adding and removing were that the p values of the variables were less than 0.05 and the VIF values were less than 10. In addition to these two important points, HAC correction was made in order not to be affected by autocorrelation and heteroscedasticity problems. It was assumed that the errors were normally distributed due to the sample size. The models that meet these conditions were ranked according to their R-squared, AIC and SC. The models in the first 5 according to this ranking are given in Table 7. The model that includes LIBOR, BACI_2, BSIS_2 as market indicators and age, DWT, shipbuilding country (Japan and China), P&I Club (UK P&I), Class (American Bureau of Shipping), ship engine manufacturer (Caterpillar) and engines rpm as the ship’s characteristics is the best model. As in the mini model, BSIS_2, which reflects the BSIS value two months before the month of sale, was found to be statistically significant in the best model. The model to be used in the comparison in the rest of the study will be called Model 2.

The resulting best 2 linear regression models obtained in the first stage, the variables used in the models, and the coefficients of these variables, p values and VIF; R^2^, adjusted R^2^,and Jarque Bera (JB) normality test probability values of the models are shown in Table 8. Since all linear regression models created had autocorrelation and heteroscedasticity problems, HAC correction [67] was made in order to evaluate p probability values properly. According to the normality test, errors are not normally distributed. However, according to the central limit theorem, it is assumed that the errors are normally distributed [67]. Although there are 1819 observations of secondhand sales, the sample size used in each regression model varies because the independent variable information for each secondhand sales observation is not complete. The sample sizes used in the model are also given in Table 8.

**Table 8:** Linear regression results for best two models.

*Model 1*				*Model 2*			
*Variable*	*Coefficient*	*P-Value*	*VIF*	*Variable*	*Coefficient*	*P-Value*	*VIF*
*C*	7.184511	0.0000	NA	*C*	7.155889	0.0000	NA
*AGE*	-0.030315	0.0000	1.467728	*AGE*	-0.030264	0.0000	1.429437
*DWT*	0.00000369	0.0004	2.067488	*DWT*	0.00000477	0.0000	1.651119
*LIBOR*	2.724869	0.0000	1.091879	*LIBOR*	1.983645	0.0000	1.168134
*BACI*	-0.0000102	0.0000	2.541593	*BSIS_2*	0.000161	0.0000	2.175036
*BSIS*	0.000151	0.0000	2.449156	*BACI_2*	-0.00000557	0.0177	2.025172
*JAPAN*	0.037464	0.0002	2.752951	*JAPAN*	0.031665	0.0009	2.881981
*CHINA*	-0.06924	0.0000	3.127340	*CHINA*	-0.075306	0.0000	3.080249
*ENGINES_RPM*	-0.002336	0.0000	3.125557	*ENGINES_RPM*	-0.001831	0.0000	1.988279
*CATERPILLAR*	-0.248724	0.0000	1.338738	*CATERPILLAR*	-0.204741	0.0000	1.107299
*UK P&I*	0.021606	0.0444	1.099398	*UK P&I*	0.025951	0.0073	1.104210
*AMERICAN BUREAU OF SHIPPING*	-0.026141	0.0356	1.116859	*AMERICAN BUREAU OF SHIPPING*	-0.022984	0.0331	1.076912
*SKULD*	0.024904	0.0442	1.156682				
*TOTAL HP MAIN ENGINE*	0.00000796	0.0308	2.249195				
*R-Squared*	0.792732			*R-Squared*	0.825909		
*Adj. R-Squared*	0.791220			*Adj. R-Squared*	0.824829		
*Autocorrelation*	Yes			*Autocorrelation*	Yes		
*Heteroskedasticity*	Yes			*Heteroskedasticity*	Yes		
*Normality(JB)Prob.*	0.0000			*Normality(JB)Prob.*	0.0000		
*Sample Size*	1796			*Sample Size*	1786		

With the regression models created in the first stage; Both the significance of the independent variables used in the study on supramax/ultramax ship prices were tested, and regression models were created for prediction. Discussions on the independent variables used in the study are given below.

- One of the independent variables frequently included in studies where individual ship sales prices are the dependent variable is *the age* variable (e.g., [6,11,23–26,55]). The expectation is that the value of the ship will decrease as its age increases. Because as the age of the ship increases, its lifespan decreases. Operating costs of new ships are lower than older ships [11], and as ships age, their earning potential decreases [25]. In the regression models created in the first stage, in line with the literature, the coefficient of the independent variable of age is negative.
- Another independent variable that is frequently included in studies where individual ship sales prices are the dependent variable is *the ship size* variable. While ship size is used as DWT, especially for dry bulk carriers and tankers (e.g., [11,23–25]); TEU is used for container ships [6,56]. Since the model is on dry bulk carriers, the ship size is expressed in DWT. As the DWT of the ship increases, the cargo carrying capacity of the ship increases, and since this increases the average freight income of the ship, the expectation regarding ship size is that there will be a positive relationship with the ship price (e.g., [7,24,25]). In the regression models created in the first stage, the coefficient of the DWT independent variable is positive, in line with the literature.
- *LIBOR* is included in the literature as the cost of capital. In studies involving LIBOR, there is no clear consensus on whether LIBOR’s effect on prices is positive or negative and whether its effect on prices is significant or not. While LIBOR was significantly and negatively effective for dry bulk carriers in the long term in the studies of Tsolakis et al. [17] and Haralambides et al. [19]; It is insignificant in the study of Thalassinos et al. [20], but it is positively effective and significant in the study of Merika et al. [55] and Kavussanos [15]. In the regression models created in the first stage, the coefficient of the LIBOR independent variable is positively effective and significant, consistent with the studies of Merika et al. [55] and Kavussanos [15].
- Albertjin et al. [11] used the Baltic Capesize Index in their study to reflect the state of the freight market and the balance of supply and demand. The researchers’ expectation is that there is a positive relationship between freight rates and ship prices, that is, if freight rates increase, ship prices will also increase, and the researchers’ results support their expectations. Junior et al. [56] used the Baltic Exchange dry index for dry bulk carriers in their study. The researchers used the logarithm Baltic Exchange dry index in the regression model they created for dry bulk carriers, and the coefficient of the variable is positive. The results of studies in the literature show that there is a positive relationship between commonly used time charter rates and secondhand ship prices [7,8,17,19,23–29,55]. In the first stage of the model, firstly, as the main variable and market indicator the value of *BACI, BPNI, BSIS* and *BHSI* in the month of the sale of the ship was added in determining the ship price. Then, the value of BACI, BPNI, BSIS and BHSI in the month of the sale of the ship and its past value were added. The main index to be focused on among the four indices is BSIS, but its interaction with other indices was also included in the model. Since the study was conducted in the supramax/ultramax segment, the coefficients of BSIS and BSIS_2 for both models are positive and consistent with the literature. The coefficients of BACI and BACI_2 are negative. This indicates that a positive change in the capesize segment negatively affects supramax/ultramax ship values. While the R-squared value for Model 1 is 0.79, the R-squared value for Model 2 is 0.83. The R-squared value, which expresses how much the independent variables explain the dependent variable, is smaller for Model 1, which takes into account the values of the Baltic Exchange indices in the sales month, while it is higher for Model 2, which takes into account the values of the Baltic Exchange indices in the month before the sales month. This indicates that the logarithmic values of the Supramax/Ultramax secondhand ship prices are better explained by Model 2.
- Another variable that is included in studies using cross-sectional data, but for which there is no consensus on its significance, is the shipbuilding country (e.g., [7,24,25,56]). In the literature, the influence of the shipbuilding country is reflected in ship quality. According to the studies of Adland et al. [25]in dry bulk carriers, Japan differs positively from other countries, and China differs negatively from other countries; According to the studies of Junior et al. [56], Japan and South Korea differ positively from other countries. In the study conducted by Merika et al. [55], where Japan, Korea, and Norway were evaluated as countries with well-known and technological shipyards; It has been concluded that the secondhand prices of ships built in these countries are positively affected. In the study conducted by Nam et al. [8], where European-made ships were taken as the basis for the idle variable; It has been concluded that among dry bulk carriers, only Chinese-made ships have a negative significance compared to European-made ships. However, there are also studies suggesting that shipbuilding countries are not effective on secondhand prices [7,28]. When the regression models created in the first stage are compared, the results are for Japanese-made ships; It appears to be consistent with the results obtained by Adland et al. [25], Junior et al. [56] and Merika et al. [55]. For Chinese-made ships; It is consistent with the study by Adland et al. [25]. Although the results are similar to the study conducted by Nam et al. [8], which claims that there is a negative significance for China; Since the study is based on European-built ships, it is considered that comparison will not be possible. In the regression analysis, the Philippines and South Korea were also added as shipbuilding countries; However, the price impact of ships built in these countries is not statistically significant. This result for South Korea is not consistent with the results of Junior et al. [56].
- *The ship’s main engine horsepower*, *engine rpm* and *ship engine manufacturer* are among the variables in the literature that try to reveal the relationship between the ship’s main engine and secondhand ship prices.While main engine horsepower ranked 11th in order of importance in the study of Adland et al. [7]; In the study of Peng et al. [28], it was found to be insignificant. While the main engine horsepower is significant in Model 1, it is not significant in Model 2. This is due to the addition of another independent variable to the model that better explains the dependent variable. Since the newly added independent variables explain the dependent variable better, the p-value of the main engine horsepower in Model 2 has increased above 0.05. While main engine horsepower is positively significant in Model 1; it is statistically insignificant in Model 2, consistent with the study of Peng et al. [28]. Main machine rpm ranks 7th in order of importance in the study of Adland et al. [7]. In the regression models created in the first stage, rpm was among the independent variables that were found to be statistically negatively significant. In the study of Adland et al. [7], ship machinery manufacturers were found to be insignificant. In the study conducted by Nam et al. [8], machine manufacturers are statistically significant. In the regression models created in the first stage, while the effect of ship main engine manufacturers variables MAN - B&W, Wartsila, Mitsubishi on secondhand ship prices was not statistically significant; Caterpillar’s effect is negatively significant.
- Among the P&I clubs that are not included in the literature but are included under the P&I club variable and whose significance we tested on ship prices with the regression models we created in the first stage, only UK P&I is statistically significant for both Model 1 and Model 2. Another P&I club, Skuld, is significant only for Model 1. This situation is due to the fact that the p value of Skuld exceeds 0.05 with the addition of variables that can better explain the dependent variable, such as the situation encountered in the main engine horsepower. The fact that the coefficients of the independent variables UK P&I and Skuld are positive indicates that the ship to be sold being insured under UK P&I or Skuld increases the price of the ship.
- Köhn [24] concluded in his study on secondhand tanker prices that the classification society variable used as an independent variable did not affect the prices. Of the ship classes added to the regression models created in the first stage, only the American Bureau of Shipping was found to be statistically significant. The negative coefficient of the variable indicates that the ship being listed under the American Bureau of Shipping has a decreasing effect on the ship’s price.
- The ship’s flag variable, which is another variable not included in the literature but included in the model, was found to be statistically insignificant in the regression models created in the first stage.
- Another variable in the literature is the general expectation regarding the crane handling capacity of the ship; It is positive because a higher crane capacity reduces the waiting time at the port, which allows the ship to make more voyages and therefore the ship earns more profit [7]. Crane handling capacity ranked 9th in order of importance in the study of Adland et al. [7]; It was also found to be statistically significant in the study of Adland et al. [25]. However, in the regression models created in the first stage, crane handling capacity was found to be statistically insignificant, unlike the literature.
- Another independent variable in the ship valuation literature is the fuel efficiency index, which also reflects the environmental friendliness of the ship. According to the results of Adland et al. [25], the fuel efficiency index gains importance especially in the period when freight rates are low; As the fuel efficiency index increases, the secondhand prices of ships also increase. Fuel efficiency index ranks 3rd after the one-year time charter rate in terms of importance in the study of Adland et al. [7]. However, the fuel efficiency index, whose current value and then its logarithm value were added to the regression models created in the first stage, is not statistically significant.

In the second stage; price prediction was made with Linear Regression, Decision Tree, Random Forest and XGBoost ML algorithms using the best two regression models determined in the first stage (Model 1 and Model 2). Since the dependent variable is logarithmic, the results obtained in the predictions are also logarithmic. While performing the performance analysis, first the dependent variable in the logarithmic structure was converted to the real price, and then the performance values were obtained. The performance analysis of the predictions is shown in Table 9.

**Table 9:** Performance analysis of the methods for the models created in the first stage.

*Method*	*Perf. Criteria*	*Linear Regression*	*Decision Tree*	*Random Forest*	*XGBoost*
*Model1*	*R* ^*2*^	0.7815	0.7916	0.8704	0.9386
	*RMSE*	5273098.58	5149908.80	4061619.71	2795287.52
	*MAE*	3499417.44	3194077.50	2484916.60	1613687.91
	*MAPE*	22.39%	21.53%	16.11%	10.43%
*Model2*	*R* ^*2*^	0.7635	0.7529	0.9281	**0.9482**
	*RMSE*	4932368.19	5041409.52	2719936.31	**2308018.31**
	*MAE*	2985164.38	2640109.35	1760184.7	**1359408.40**
	*MAPE*	20.22%	18.28%	12.81%	**9.88%**

According to the results in Table 9, XGBoost gives the best result in the performance analysis comparison; Random Forest, Decision Tree, and Linear Regression follow XGBoost, respectively. For all performance values, Model 2 gives better results than Model 1. A MAPE value below 10% indicates that the model has high predictive accuracy, acceptable performance, and high predictive power. While the MAPE value for Model 2 is below 10%, it is above 10% for Model 1. This shows that Model 2 is a usable model for prediction. The opposite is true for Model 1.

In the third section, where the main purpose is to examine the effect of the extracted independent variables on prediction; Engine rpm, main engine horsepower, P&I club, machine manufacturer, and class variables were removed from the regression models created in the first stage. The results of the new regression models are shown in Table 10. The performance analysis results obtained as a result of re- predicting the new models created with Linear Regression, Decision Tree, Random Forest, and XGBoost ML algorithms are shown in Table 11.

**Table 10:** Linear regression results for best two models.

*Model 1*				*Model 2*			
*Variable*	*Coefficient*	*P-Value*	*VIF*	*Variable*	*Coefficient*	*P-Value*	*VIF*
*C*	6.799944	0.0000	NA	*C*	6.791589	0.0000	NA
*AGE*	-0.030581	0.0000	1.137328	*AGE*	-0.030873	0.0000	1.088673
*DWT*	0.00000752	0.0000	1.074209	*DWT*	0.00000763	0.0000	1.054412
*LIBOR*	2.639353	0.0000	1.070594	*LIBOR*	1.944537	0.0000	1.121347
*BACI*	-0.0000102	0.0000	2.440329	*BSIS_2*	0.000162	0.0000	1.978710
*BSIS*	0.000152	0.0000	2.409760	*BACI_2*	-0.00000676	0.0034	1.925521
*JAPAN*	0.029063	0.0070	2.838454	*JAPAN*	0.027187	0.0075	2.940351
*CHINA*	-0.076964	0.0000	2.952378	*CHINA*	-0.085097	0.0000	3.181282
*R-Squared*	0.785018			*R-Squared*	0.818433		
*Adj. R-Squared*	0.784187			*Adj. R-Squared*	0.817727		
*Autocorrelation*	Yes			*Autocorrelation*	Yes		
*Heteroskedasticity*	Yes			*Heteroskedasticity*	Yes		
*Normality(JB)Prob.*	0.0000			*Normality(JB)Prob.*	0.0000		
*Sample Size*	1819			*Sample Size*	1809		

**Table 11:** Performance analysis of the methods for the models created in the third stage.

*Method*	*Perf. Criteria*	*Linear Regression*	*Decision Tree*	*Random Forest*	*XGBoost*
*Model1*	*R* ^*2*^	0.8108	0.8568	0.9043	0.9288
	*RMSE*	4565067.08	3970990.42	3246605.71	2799764.801
	*MAE*	3212173.15	2579850.72	1921405.52	1519849.76
	*MAPE*	21.68%	18.43%	12.83%	10.10%
*Model2*	*R* ^*2*^	0.5517	0.8942	0.9398	**0.9490**
	*RMSE*	7958002.81	3866763.66	2915657.33	**2682627.35**
	*MAE*	3411627.79	2367560.02	1757350.8	**1473897.56**
	*MAPE*	19.33%	15.59%	11.31%	**9.35%**

According to the results in Table 10, the XGBoost method gives the best result in the performance analysis comparison for all models. XGBoost is followed by Random Forest, Decision Tree and Linear Regression. In the comparison of models based on the XGBoost method, Model 2 gave the best result according to all performance criteria. As in the previous performance analysis, the MAPE value is below 10% for Model 2 and above 10% for Model 1. This shows that the simplified Model 2 is still a usable model for prediction. The closeness of the results obtained in the second stage and the third stage allows the use of simplified models in prediction.

In the fourth stage; Prediction was made with the regression models obtained in the third stage with the added 2023 data. The results of the predictions obtained with the models created using sales data for the period between August 2005 and December 2023 are shown in Table 12.

**Table 12:** Performance analysis of the methods for the models formed by adding 2023 data to the data set of the models created in the third stage.

*Method*	*Perf. Criteria*	*Linear Regression*	*Decision Tree*	*Random Forest*	*XGBoost*
*Model1*	R^2^	0.8428	0.8549	0.9158	0.9307
	RMSE	4300674.91	4132518.74	3146993.16	2856722.43
	MAE	2997949.45	2628416.82	1785055.89	1488747.37
	MAPE	20.20%	18.59%	12.21%	9.60%
*Model2*	R^2^	0.7920	0.9247	0.9565	**0.9676**
	RMSE	5029477.03	3026666.07	2299002.46	**1984335.65**
	MAE	2913137.84	2151023.15	1544016.59	**1282572.71**
	MAPE	18.79%	16.15%	10.67%	**8.70%**

**Fig 3:**
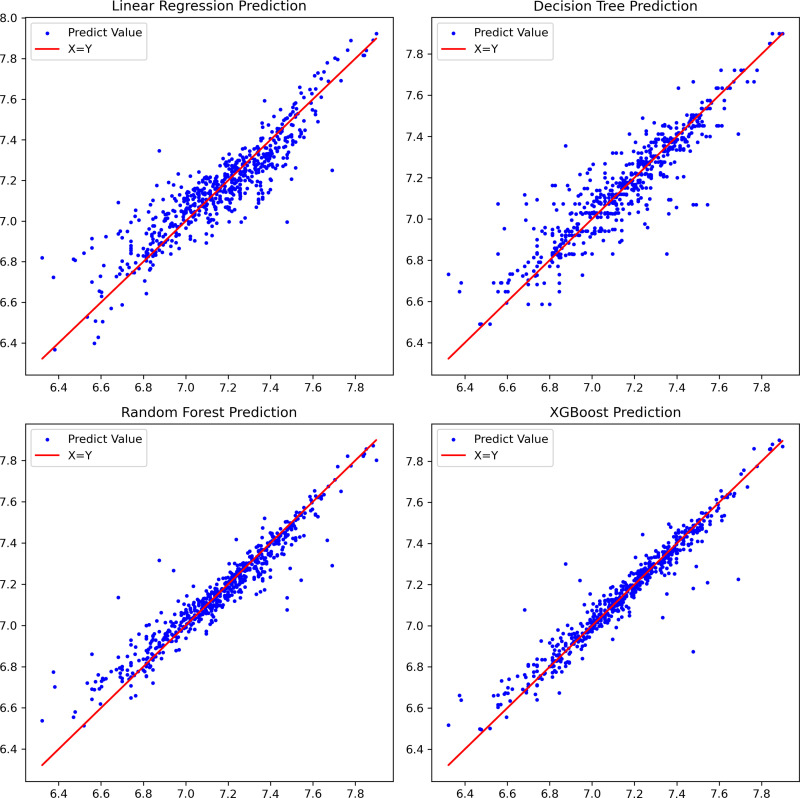
The results of Model 1.

**Fig 4:**
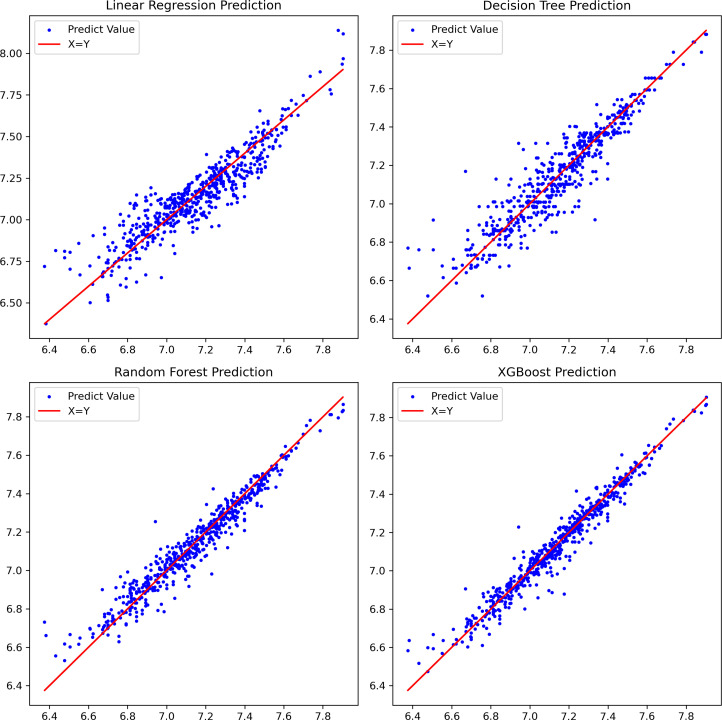
The results of Model 2.

According to the performance analysis in Table 12, the XGBoost method gives the best results as in other stages. XGBoost is followed by Random Forest, Decision Tree, and Linear Regression, respectively. In the comparison of models based on the XGBoost method, Model 2 gave the best results for R^2^, RMSE, MAE, MAPE values. Linear Regression, Decision Tree, Random Forest and XGBoost results are presented in Fig 3 for Model 1. Linear Regression, Decision Tree, Random Forest, and XGBoost results are presented in Fig 4 for Model 2. The performance analysis results and graphs clearly reveal the superiority of Model 2 over Model 1. Consistent with Adland et al. [7] XGBoost stands out as the best prediction method among the methods used.

In the last stage, the MAPE value of the model was found to be 9.6% in the prediction made using the XGBoost method with Model 1. This value is an acceptable value for the use of the model in prediction. In addition, the MAPE value of Model 2 is 8.7%, which clearly shows that Model 2 will yield much better results than Model 1 when used in prediction. When the R-squared values are considered, the R-squared value of Model 1 is 0.93, while the R-squared value of Model 2 is 0.97. When Model 2 is examined in terms of R-squared values, it is much better than Model 1. In addition, according to RMSE and MAE values, Model 2 is ahead of Model 1. When all the results obtained are combined, it is clearly seen that the model using the Baltic Exchange indices of the months before the month of sale produces much better results than the model using the Baltic Exchange indices of the month of sale. The fact that the model can be created with data that can be easily obtained from the market and that XGBoost can be easily applied in programs such as Python allows the model to be easily applied by units in the market (shipowners, insurers, banks). When considered from the perspective of shipowners; A shipowner may need a ship valuation before making purchasing, selling and financial decisions; and can make a decision according to the value he will obtain with this model. The fact that the model uses the past values of the Baltic Exchange indices also benefits the shipowner in the ship purchase and sale process. Model 2 uses the values of BSIS and BACI 2 months ago; this allows the value that the ship will receive in two months to be determined from the current month.

Especially when making investment decisions, when calculating investment returns; Calculating the accurate ship values allows for a comprehensive assessment of the return on investment and for investment decisions to be made accurately. Another point that can be taken into consideration by shipowners and financial institutions is risk management. Accurate ship valuation allows shipowners and financial institutions to manage risks properly. Accurate ship valuation allows financiers to support the right investments. The model also contributes to brokers in ship valuations. They can easily evaluate ships with the data sets they 1create. Insurers can benefit from this study when calculating the value of the asset they will insure and determining the insurance value according to this value.

## 6. Conclusion, limitations and future research

The studies related to both ship valuation and Machine Learning methods have been increasing day by day in the literature, likewise, nowadays the importance of artificial intelligence is more increasing each day. This study focuses on the ship valuation by using ML methods; therefore, the aim of this study is to propose a ship valuation model with high accuracy that will be available to interested parties in the market using ML methods, based on linear regression models created with variables determined by testing their significance on the basis of linear regression for dry bulk carriers in the Supramax/Ultramax ship segment.

This study is designed structured in four stages: *(i)* Considering the data set, *Linear Regression* analyses were conducted where the logarithm of ship sales prices was the dependent variable. In the analysis, variables were added and removed depending on the *p-value*. *The 2 best models* were determined according to *R*^*2*^*, AIC, and SC criteria*. One of the models considers the Baltic Exchange indices in the sales month as independent variables; the other considers the Baltic Exchange indices in the months before the sales month and the Baltic Exchange indices in the sales month as independent variables. *(ii)* The determined 2 regression models were computed to predict ship’ prices by using the ML algorithms namely *Linear Regression*, *Decision* Tree, *Random Forest*, and *XGBoost*. The ship’s prices in the 30% test data were predicted by these models so obtained values of *R*^*2*^, *RMSE*, *MAE*, and *MAPE* performance analysis to compare the models. According to the comparing results, the *XGBoost* method is the most effective among these methods in *Model2. (iii)* This stage created the simplified models is to find out the effect of the extracted independent variables on the prediction. The results of this stage compared to the result of the second stage are obtained to close to each other allows the use of simplified models in the prediction. *(iv)* The simplified models were performed with expanded data, it is observed that *XGBoost* method is the best performing method among all ML methods according to the results of the performance anaylsis with the MAPE value as %8.7.

The results show that for Model 1 using the Baltic Exchange indices of the month of sale, the market indicators: LIBOR, BACI, BSIS, and the ship’s characteristics: age, DWT, shipbuilding country (Japan and China), P&I Club (UK P&I and Skuld), Class (American Bureau of Shipping), ship engine manufacturer (Caterpillar), engines rpm and total horsepower main engine are significant for Supramax/Ultramax ship prices; while for Model 2 using the Baltic Exchange indices of the months before the month of sale, the market indicators: LIBOR, BACI_2, BSIS_2, and the ship’s characteristics: age, DWT, shipbuilding country (Japan and China), P&I Club (UK P&I), Class (American Bureau of Shipping), ship engine manufacturer (Caterpillar) and engines rpm are significant for Supramax/Ultramax ship prices. On the other hand; in the fourth stage, while the *R*^*2*^ value of *Model 2* for *linear regression* was 0.79, this value has been increased to 0.97 with *XGBoost*, which represents an improvement of 23%, so, this clearly shows that ML is much better than traditional methods. In general, a *MAPE* value of less than 10% indicates that the model produces very good results. The values of *MAPE* are below 10% for almost all models created; this value have dropped to 8.7% for the re-predicted *Model 2* with the addition of 2023 data. Hence, proposed methods smoothly work for ship valuation.

This research introduces, as a novel contribution to the literature, the utilization of the ML approach for ship valuation of in shipping industry. This study contributes to the literature as summarized as follows: *(i)* With this study it is the first time the ship valuation is carried out in the Supramax/Ultramax ships segment by ML methods such *Linear Regression*, *Decision* Tree, *Random Forest*, and *XGBoost*. *(ii)* In this study, unlike the studies in the literature, in addition to the values of the Baltic Exchange indices in the month of the ship sale, the values of the Baltic Exchange indices before the month of the ship sale were also added to the models created. The results show that Model 2, which includes the Baltic Exchange indices of the months before the month of the ship sale, is much better than Model 1 in predicting the prices of Supramax/Ultramax ships. Making predictions with models that include the Baltic Exchange indices of the months before the month of the ship sale also provides the interested parties with the opportunity to make future predictions. *(iii)* In the study, *P&I* and *Flag* were firstly used as the independent variables. While the *P&I* was statistically positively significant, the *Flag* was found to be statistically insignificant. *(iv)* The proposed methodology is one of the methods that obtains high-accuracy solutions for the Supramax/Ultramax ships segment with data that is easily accessible in the market.

Accurate ship valuation assists decision makers in making strategic decisions, optimizing purchasing and sales timing, managing risk and long-term planning. Ships, which are capital-intensive assets, are among the items to which a significant portion of capital is allocated, especially in maritime businesses where shipowners are at the forefront. Accurate valuation of ships will also contribute to businesses effectively determining their capital structures. The model in the study is shaped by Baltic Exchange indexes and LIBOR, as well as easily accessible information such as ship age, DWT, and shipbuilding country. Future ship values can be determined by making predictions for Baltic Exchange indexes and LIBOR. These predictions can contribute to businesses determining possible financial risks. Since the variables used in the simplified model can be easily accessed from shipbroker reports, it is thought that this proposed model can be easily used by relevant parties in purchasing and sales transactions, financing and credit provision, strategic planning, insurance transactions, accounting and financial reporting, investment evaluations, acquisitions and mergers, leasing, and operational agreements. Consequently, this research has the potential to provide valuable insights for academics and industry professionals seeking to enhance and utilize their approach in ship valuation.

Regarding limitations and future research, the validity of models created with simplified model logic for other ship types, such as containers and tankers, especially other dry cargo segments, can be investigated. The main key point in creating a model with the same logic as the model to be created here is to add the values of the Baltic Exchange indices in the month of sale as well as the values before the month of sale to the model. Additionally, models can be created for sub-segments of other ship types using ship type-specific indices. Although order/fleet, newbuilding price, and scrap values are available in the literature, they were not accessible to the researchers, so they were not included in the regression models established in the first stage, and the effects of the variables on Supramax/Ultramax ships could not be examined. These variables can be added to comprehensive models to be built on Supramax/Ultramax ships and can be added to the models according to their statistical significance.

## References

[1] UNCTAD. Review of Maritime Transport 2023 (UNCTAD/RMT/2023) [Internet]. New York: United Nations Conference on Trade and Development; 2023 [cited 2024 Jan 31]. Available from: https://unctad.org/publications-search?f%5B0%5D=product%3A393

[2] UNCTAD. Review of Maritime Transport 2022 (UNCTAD/RMT/2022) [Internet]. New York: United Nations Conference on Trade and Development; 2022 [cited 2024 Jan 31]. Available from: https://unctad.org/publications-search?f%5B0%5D=product%3A393

[3] Kavussanos MG, Tsouknidis DA, Visvikis ID. Freight derivatives and risk management in shipping. Oxford, UK: Routledge; 2021 Apr 29.

[4] Stopford M. Maritime economics 3e. Routledge; 2008 Dec 19.

[5] Canca AY. Gemi finansman kredi sözleşmelerinde değerleme ve yeniden değerleme riski. J Eta Maritime Sci. 2015;3(2):127-32. doi: 10.5505/jems.2015.14633

[6] Mietzner A. Developing a dynamic vessel valuation method based on real market transactions. In: InHSBA Handbook on Ship Finance. Berlin, Heidelberg: Springer Berlin Heidelberg; 2014 Oct 31. p. 165–82. doi: 10.1007/978-3-662-43410-9_10

[7] Adland R, Jia H, Harvei HC, Jørgensen J. Second-hand vessel valuation: an extreme gradient boosting approach. Marit Policy Manag. 2023 Jan 2;50(1):1–18. doi: 10.1080/03088839.2021.1969601

[8] Nam H-S, De Alwis N, D’agostini E. Determining factors affecting second-hand ship value: linkages and implications for the shipbuilding industry. WMU J Marit Aff. 2022 Dec;21(4):493–517. doi: 10.1007/s13437-022-00272-4

[9] Syriopoulos T, Tsatsaronis M, Karamanos I. Support vector machine algorithms: An application to ship price forecasting. Comput Econ. 2021;57(1):55-87. doi: 10.1007/s10614-020-10032-2

[10] International Valuation Standards Council. International valuation standards 2017. 1st ed. London: International Valuation Standards Council; ISBN 978-0-9931513-0-9. 2017.

[11] Albertijn S, Drobetz W, Johns M. Maritime investment appraisal and budgeting. In: The international handbook of shipping finance: theory and practice. London: Palgrave Macmillan UK; 2016 Nov 12. p. 285–313.

[12] Mayr D. Valuing vessels. In: HSBA Handbook on Ship Finance. Berlin, Heidelberg: Springer Berlin Heidelberg; 2014 Oct 31. p. 141–63.

[13] Kavussanos MG. Comparisons of volatility in the dry-cargo ship sector: Spot versus time charters, and smaller versus larger vessels. J Transp Econ Policy. 1996;30(1):67–82.

[14] Kavussanos MG. Price risk modelling of different size vessels in the tanker industry using autoregressive conditional heteroskedastic (ARCH) models. Logist Transp Rev 1996;32(2):161.

[15] Kavussanos MG. The dynamics of time-varying volatilities in different size second-hand ship prices of the dry-cargo sector. Appl Econ. 1997;29(4):433–43. doi: 10.1080/000368497326930

[16] Glen DR, Martin BT. Conditional modelling of tanker market risk using route specific freight rates. Maritime Policy & Management. 1998;25(2):117–28. doi: 10.1080/03088839800000023

[17] Tsolakis SD, Cridland C, Haralambides HE. Econometric modelling of second-hand ship prices. Marit Econ Logist. 2003;5(4):347–77. doi: 10.1057/palgrave.mel.9100086

[18] Alizadeh AH, Nomikos NK. The price-volume relationship in the sale and purchase market for dry bulk vessels. Marit Policy Manag. 2003;30(4):321–37. doi: 10.1080/0308883032000145627

[19] Haralambides HE, Tsolakis SD, Cridland C. Econometric modelling of newbuilding and secondhand ship prices. Res Transp Econ. 2005;12(1):65–105. doi: 10.1016/S0739-8859(04)12003-9

[20] Thalassinos EI, Politis E. Valuation model for a second-hand vessel: Econometric analysis of the dry bulk sector. J Glob Bus Technol. 2014;10(1).

[21] Wright G. Market fundamentals, market sentiment and second-hand ship prices. Int J Transp Econ. 2005;XXXII(1):1000–9.

[22] Adland R, Jia H, Strandenes S. Asset bubbles in shipping? An analysis of recent history in the drybulk market. Marit Econ Logist. 2006;8:223–33. doi: 10.1057/palgrave.mel.9100162

[23] Adland R, Koekebakker S. Ship Valuation Using Cross-Sectional Sales Data: A Multivariate Non-Parametric Approach. Marit Econ Logist. 2007;9:105–18. doi: 10.1057/palgrave.mel.9100174

[24] Köhn S. Generalized additive models in the context of shipping economics [dissertation]. Leicester: University of Leicester; 2008.

[25] Adland R, Cariou P, Wolff F-C. Does energy efficiency affect ship values in the second-hand market?. Transportation Research Part A: Policy and Practice. 2018;111:347–59. doi: 10.1016/j.tra.2018.03.031

[26] Lim SS, Lee KH, Yang HJ, Yun HS. Panamax second-hand vessel valuation model. J Navig Port Res. 2019;43(1):72–8. doi: 10.5394/KINPR.2019.43.1.72

[27] Adland R, Köhn S. Semiparametric valuation of heterogeneous assets. In: Asset Intelligence through Integration and Interoperability and Contemporary Vibration Engineering Technologies: Proceedings of the 12th World Congress on Engineering Asset Management and the 13th International Conference on Vibration Engineering and Technology of Machinery. Springer International Publishing; 2019. p. 23-30. Available from: doi: 10.1007/978-3-319-95711-1_3

[28] Peng WH, Adland R, Yip TL. Investor domicile and second-hand ship sale prices. Maritime Policy & Management. 2021;48(8):1109–23. doi: 10.1080/03088839.2020.1839684

[29] Kim D, Choi J-S. Development of Ship Valuation Model by Neural Network. J Korean Soc Mar Environ Saf. 2021;27(1):13–21. doi: 10.7837/kosomes.2021.27.1.013

[30] Kalouptsidi M. Time to build and fluctuations in bulk shipping. American Economic Review. 2014;104(2):564–608. doi: 10.1257/aer.104.2.564

[31] Müller AC, Guido S. Introduction to machine learning with Python: a guide for data scientists. Sebastopol (CA): O’Reilly Media, Inc.; 2016.

[32] Géron A. Hands-on machine learning with Scikit-Learn, Keras, and TensorFlow. Sebastopol (CA): O’Reilly Media, Inc. 2022.

[33] United States Department of Agriculture. Ocean bulk vessel fleet size by capacity [Internet]. 2024 [cited 2024 Sep 20]. Available from: https://agtransport.usda.gov/Bulk/Ocean-bulk-vessel-fleet-size-by-capacity/a2id-9qs9

[34] AXSData. Bulk carriers fleet growth: a decade in review and 2025 outlook [Internet]. 2024 [cited 2024 Sep 20]. Available from: https://public.axsmarine.com/blog/bulk-carriers-fleet-growth-a-decade-in-review-and-2025-outlook

[35] AXSData. New records for dry bulk flows in 2023 [Internet]. 2024 [cited 2024 Sep 20]. Available from: https://public.axsmarine.com/blog/new-records-for-dry-bulk-flows-in-2023

[36] Baltic Exchange. Guide to Baltic Code 2020 & introductory guide to modern shipping [Internet]. 2020 [cited 2024 Mar 16]. Available from: https://www.balticexchange.com/content/dam/balticexchange/consumer/documents/data-services/documentation/ocean-bulk-guides-policies/2020_BalticCode.pdf

[37] CW Kellock & Co. Sales history [Internet]. 2024 [cited 2024 Feb 2]. Available from: https://cwkellock.com

[38] Charemza W, Gronicki M. An econometric model of world shipping and shipbuilding. Marit Policy Manag. 1981;8(1):21–30. doi: 10.1080/03088838100000019

[39] Beenstock M. A theory of ship prices. Marit Policy Manag. 1985;12(3):215–225. doi: 10.1080/03088838500000028

[40] Böhm-Bawerk EV. The positive theory of capital. Smart W, translator. New York: G. E. Stechert & Co.; 1930.

[41] Usher D Traditional capital theory. Rev Econ Stud. 1965;32(2):169–86.

[42] Beenstock M, Vergottis A. An econometric model of the world market for dry cargo freight and shipping. Appl Econ. 1989;21(3):339–56. doi: 10.1080/758522551

[43] Beenstock M, Vergottis A. An econometric model of the world tanker market. J Transp Econ Policy. 1989;263–80.

[44] Beenstock M, Vergottis A. The interdependence between the dry cargo and tanker markets. Logist Transp Rev. 1993;29(1):3.

[45] Hale C, Vanags A. The market for second-hand ships: some results on efficiency using cointegration. Marit Policy Manag. 1992;19(1):31–9. doi: 10.1080/03088839200000003

[46] Kavussanos MG, Alizadeh AH. Efficient pricing of ships in the dry bulk sector of the shipping industry. Marit Policy Manag. 2002;29(3):303–30. doi: 10.1080/03088830210132588

[47] Glen DR. The market for second-hand ships: Further results on efficiency using cointegration analysis. Marit Policy Manag. 1997;24(3):245–60. doi: 10.1080/03088839700000029

[48] Ådland AO, Koekebakker S. Market efficiency in the second-hand market for bulk ships. Marit Econ Logist. 2004;6(1):1–15. doi: 10.1057/palgrave.mel.9100092

[49] Alizadeh AH, Nomikos NK. Investment timing and trading strategies in the sale and purchase market for ships. Transportation Research Part B: Methodological. 2007;41(1):126–143. doi: 10.1016/j.trb.2006.04.002

[50] Engelen S, Dullaert W, Vernimmen B. Market efficiency within dry bulk markets in the short run: a multi-agent system dynamics Nash equilibrium. Marit Policy Manag. 2009;36(5):385-396. doi: 10.1080/03088830903187135

[51] Syriopoulos T, Roumpis E. Price and volume dynamics in second-hand dry bulk and tanker shipping markets. Marit Policy Manag. 2006;33(5):497–518. doi: 10.1080/03088830601020729

[52] Merikas AG, Merika AA, Koutroubousis G. Modelling the investment decision of the entrepreneur in the tanker sector: choosing between a second-hand vessel and a newly built one. Maritime Policy & Management. 2008;35(5):433–447. doi: 10.1080/03088830802352053

[53] Lun YV, Quaddus MA. An empirical model of the bulk shipping market. Int J Shipping Transp Logist. 2009;1(1):37-54.

[54] Adland R, Jia H. Shipping market integration: The case of sticky newbuilding prices. Marit Econ Logist. 2015;17:389–398. doi: 10.1057/mel.2014.35

[55] Merika A, Merikas A, Tsionas M, Andrikopoulos A. Exploring vessel-price dynamics: the case of the dry bulk market. Marit Policy Manag. 2019;46(3):309–329. doi: 10.1080/03088839.2018.1562246

[56] Junior FCP, Fiasca RB, Assis LF. Shipbuilder country and second-hand price. Int J Shipping Transp Logist. 2020;12(5):426-444. doi: 10.1504/IJSTL.2020.109886

[57] Pruyn JFJ, van de Voorde E, Meersman H. Second hand vessel value estimation in maritime economics: A review of the past 20 years and the proposal of an elementary method. Marit Econ Logist. 2011;13:213–236. doi: 10.1057/mel.2011.6

[58] Ross HH. Second-hand price volatility of green ships: an empirical analysis across main shipping segments. Diskussionspapier. 2019;182.

[59] Koray M, Cetin O. A combined qualitative ship valuation estimation model. WMU J Marit Aff. 2019;19(2):205-217. doi: 10.1007/s13437-020-00202-2

[60] Baltic Handysize (BHSI) historical data. [Internet]. Investing.com; 2024 Jan 10 [cited 2024 Jan 31]. Available from: https://www.investing.com/indices/baltic-handysize-historical-data

[61] Investing. Baltic Capesize (BACI) historical data [Internet]. Investing.com; 2024 Jan 10 [cited 2024 Jan 31]. Available from: https://www.investing.com/indices/baltic-capesize-historical-data

[62] Investing. Baltic Panamax (BPNI) historical data [Internet]. Investing.com; 2024 Jan 10 [cited 2024 Jan 31]. Available from: https://www.investing.com/indices/baltic-panamax-historical-data

[63] Investing. Baltic Supramax (BSIS) historical data [Internet]. Investing.com; 2024 Jan 10 [cited 2024 Jan 31]. Available from: https://www.investing.com/indices/baltic-supramax-historical-data

[64] Global-Rates. American Dollar LIBOR rates [Internet]. Global-Rates.com; 2024 Jan 10 [cited 2024 Jan 31]. Available from: https://www.global-rates.com/en/interest-rates/libor/american-dollar/.

[65] IHS. Ship data for bulker [Internet]. IHS.com; 2021 Dec 31 [cited 2024 Jan 31]. Available from: https://maritime.ihs.com/Account2/Index

[66] Hastie T, Tibshirani R, Friedman JH. The elements of statistical learning: data mining, inference, and prediction. Vol. 2. New York: Springer; 2009.

[67] Gujarati D. Econometrics by example. London: Bloomsbury Publishing; 2014.

[68] Kumar A. Learning predictive analytics with Python. Birmingham: Packt Publishing Ltd; 2016.

[69] Bowles M. Machine learning in Python: essential techniques for predictive analysis. Hoboken: John Wiley & Sons; 2015.

[70] Wade C, Glynn K. Hands-on gradient boosting with XGBoost and scikit-learn: perform accessible machine learning and extreme gradient boosting with Python. Birmingham: Packt Publishing Ltd; 2020.

[71] Quinto B. Next-generation machine learning with Spark: covers XGBoost, LightGBM, Spark NLP, distributed deep learning with Keras, and more. Berkeley: Apress; 2020.

[72] Chen T, Guestrin C. XGBoost: a scalable tree boosting system. In: Proceedings of the 22nd ACM SIGKDD international conference on knowledge discovery and data mining. San Francisco, CA, USA. 2016 Aug. p. 785–94.

[73] Brownlee J. XGBoost with Python: Gradient boosted trees with XGBoost and scikit-learn. Machine Learning Mastery; 2016.

